# Improving the antimicrobial activity of RP9 peptide through theoretical and experimental investigation

**DOI:** 10.1016/j.bbrep.2025.101953

**Published:** 2025-02-15

**Authors:** Mahya Anahid, Karim Mahnam, Behnaz Saffar

**Affiliations:** a Department of Genetics, Faculty of Science, Shahrekord University, Shahrekord, Iran; b Department of Biology, Faculty of Science, Shahrekord University, Shahrekord, Iran; c Biotechnology Research Institute, Shahrekord University, Shahrekord, Iran

**Keywords:** Antimicrobial peptide, RP9, Molecular dynamic simulation, Mutation, Octanol, MIC

## Abstract

Future threats to humanity may stem from the rise of antimicrobial resistance, which has compromised the effectiveness of existing antibiotics. Antimicrobial peptides possess the ability to directly eliminate pathogens and cancer cells, generally without the development of resistance. Among these peptides is RP9 (RGSALTHLP), derived from the white blood cells of crocodiles. In this research, three mutations were initially designed: LR-mut (RGSALTHLR), KR-mut (RGSAKTHLR), and WP-mut (RGSAWTHLP). The physicochemical characteristics of these peptides were assessed, revealing that KR-mut exhibited the most favorable biophysical properties. Subsequently, twenty molecular dynamics simulations were conducted for all peptides in pure water and at four different octanol concentrations (30 %, 50 %, 70 %, and 100 %) to evaluate their biophysical attributes. The findings from the 4000 ns molecular dynamics simulations revealed that the KR-mut exhibited reduced values of RMSD, the radius of gyration, solvent accessible surface area, and RMSF, while simultaneously showing an increased number of hydrogen bonds and interactions with water molecules. This peptide also showed the lowest free energy of solvation and the highest solubility across various octanol concentrations compared to the other peptides. The results obtained from the biophysical assessments and molecular dynamics simulations were consistent, resulting in the conclusion that KR-mut is expected to exhibit superior antibacterial activity compared to both the other mutated peptides and the wild type peptides. These theoretical findings were validated through experimental minimum inhibitory concentration (MIC) tests on gram-negative *Escherichia coli* and gram-positive *Staphylococcus aureus*. The outcomes of this study suggest that molecular dynamics simulations can effectively predict changes in the bactericidal efficacy of peptides at varying octanol concentrations, potentially enhancing the speed and efficiency of antimicrobial peptide design while reducing associated costs.

## Highlights

• “RGSAKTHLR” peptide has more antibacterial properties than native RP9 peptide.
• The experimental MIC test verified the theoretical study.
• The MD simulations can predict changes in the bactericidal efficacy of peptides.

## Graphical abstract

**Figure:**
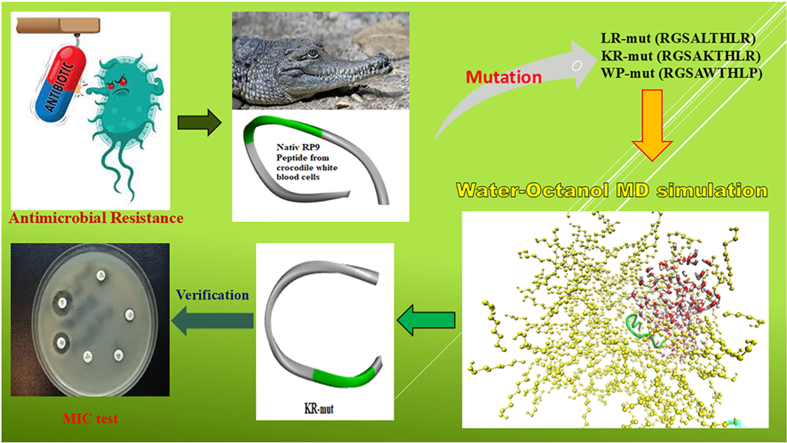

## 1 Introduction

The rise of antibiotic-resistant forms of infectious diseases is making effective treatment more and more complex, and these diseases are directed toward a global problem. With a molecular weight of typically less than 10 kDa, antimicrobial peptides (AMPs) are a varied class of naturally occurring compounds produced by living organisms as their first line of defense [1]. They also possess positive and basic residues of varying lengths and exhibit amphiphilicity [2]. These peptides are probably safe for mammalian cells and work by permeabilizing pathogen cell membranes. The inner monolayer of the plasma membrane of human cell membranes mostly has zwitterionic phospholipids, which block cationic AMPs from interacting with the plasma membrane. Furthermore, the increased levels of phosphatidylcholine and sphingomyelin in the outer monolayer produce a tightly packed surface that prevents peptides from binding to the lipid bilayer [3].

The extensive range of research on antimicrobial peptides has increased for two reasons: firstly, bacteria have developed resistance to antibiotics. Second, the improvement of antibiotics is complicated and limited [4]. Information on the molecular basis of the mechanism of antimicrobial peptide function facilitates the designing of new drugs based on these peptides. There are numerous models of antimicrobial peptide functions [5]. Three of them start from the same conformation, with the peptides associating with the bacterial membrane as the barrel-stave model, the carpet model (also inflicting pore formation), and the form of the toroidal pore, which creates pores wherein peptides and lipids are blended and peptides bend into the pore continuously from the surface of the membrane. Given the heterogeneity of the structure and size of AMPs, each peptide may want to use one or more of these fashions in its interactions with membranes [[6], [7], [8]]. It has additionally been mentioned that antimicrobial peptides bind to the membrane via the interactions of positive charges of arginine or lysine residues with negative charges at the membrane surface. During these interactions, the side chain of aromatic residues is oriented and enters the membrane [9]. The experimental look at the interactions of antimicrobial peptides with membranes has a few obstacles, such as the complex structure of the membrane, the asymmetry of the membrane surface, and the variation of the structure of the membrane while labeled with fluorescence probes [10].

Crocodile blood has long been used as a conventional medicinal drug in many Asian locations to treat diseases. In addition, in vivo or in vitro research showed that crocodile blood may have numerous valuable applications, which include antimicrobial, antiviral, antioxidant, anti-inflammatory, antitumor, antianaemia, and wound recuperation applications. White blood cell extract of freshwater *Crocodylus siamensis* is a natural supply that contains many bioactive peptides and indicates many biological properties, including antimicrobial, antioxidant, anti-inflammatory, and anticancer activities [11].

The RP9 peptide (RGSALTHLP), derived from the white blood cell extract of freshwater *Crocodylus siamensis*, has antimicrobial and anticancer properties. The ID code of this peptide in the antimicrobial peptide database (https://aps.Unmc.Edu/database/peptide) is AP02691. The effects of this peptide on bacterial cells had been observed using a scanning electron microscope. The difference between this peptide and other peptides is that the RP9 peptide is present within the crocodile blood. Most of the other peptides are found within the stratum corneum or the mucosal layers of the skin. This suggests that the toxicity of this peptide to erythrocytes is trivial and that it could be used as an injectable drug. In addition, this peptide has a short sequence, and the cost of its preparation is mediocre [12].

Molecular dynamics simulation (MD) offers a physically based foundation for precisely describing the motions of atoms inside molecular systems. Currently, MD and experimental methods have been used in the examination of protein and bacterial cell envelope structure and function [13]. MD can be used to study the mechanisms of action of antimicrobial peptides [14]. In addition, explicit solvent MD is useful for predicting biomembrane penetrating peptides, defining their molecular mechanism of passive penetration, and exactly assessing the energetic and structural favorites of the peptide structures in aqueous solutions [15].

Although 1-octanol can't form stable structures consisting of bilayers or micelles, which might be typical of lipid solutions, it can successfully mimic susceptible weak polar solvents and some of the properties of biologically appropriate structures. It is commonly used for this cause [16], and it could be a surrogate for complicated phospholipids that comprise bilayer membranes [17]. Also, the water/octanol partition coefficient is a broadly generic quantity of lipophilicity, has been used as a simple and ideal model for determining the lipophilicity of molecules, and is an excellent membrane-mimetic surroundings model for the water/membrane interface [14,18,19]. This enables us to comprehend the process by which bioactive molecules penetrate biological membranes, a crucial attribute for their effectiveness.

## 2 Method

This work was conducted in two sections. Within the first section, the wild type and mutated RP9 peptides have been used for bioinformatics studies to evaluate their antibacterial properties. Then these peptides were simulated with the Gromacs 2020 package [20] at different concentrations of octanol to predict the biophysical properties of these peptides. In the second section, experimental research has been performed on the wild type RP9 and KR-mut (RGSAKTHLR) peptides to evaluate the theoretical results.

### 2.1 Theoretical methods

At first, the wild type RP9 peptide (RGSALTHLP) structure was built using the Pepfold server (https://bioserv.rpbs.univ-paris-diderot.fr/services/PEP-FOLD3/) as a helix [21] and saved in PDB format. The antimicrobial peptides are amphipathic or cationic (positive net charge at physiological pH), have sequence lengths between five and 40 residues, are soluble in water and partially hydrophobic, and are rich in lysine and arginine residues [[22], [23], [24]]. Nguyen et al. demonstrated that the binding of positively charged peptides rich in arginine to neutral bilayers is a crucial process for the transport of these peptides into the interior of the cell [25]. Vargas et al. showed that the effectiveness of membrane penetration depends on the number of guanidine moieties [26]. Arginine and lysine residues are positive and bulky, which helps electrostatic and hydrophobic interactions of the peptide with the membrane. The peptides, rich in Arg and Lys residues, effectively traverse the high free energy barrier of hydrophobic cellular membranes [14,27]. Therefore, three mutations were created into the wild type RP9 (RGSALTHLP) peptide that includes: LR-mut (RGSALTHLR) via transformation of Pro9 to Arg, KR-mut (RGSAKTHLR) via transformation of Leu5 to Lys and Pro9 to Arg to increase the positive charge of the wild type peptide, and WP-mut (RGSAWTHLP) by changing Leu5 to Trp to increase the hydrophobicity of the wild type RP9 peptide [28].

Then, some biophysical properties such as the GRAVY parameter (https://www.gravy-calculator.de/), hydrophobicity (https://www.peptide2.com/Npeptide_hydrophobicity_hydrophilicity.php), dipole moment (https://dipole.protopedia.org/), amphiphilicity (https://webs.iiitd.edu.in/raghava/toxinpred/design.php), the octanol scale or free energies of the transfer peptides from water to n-octanol (https://blanco.biomol.uci.edu/hydrophobicityscales.html), cell-penetrating of peptides (CP), molecular weight (MW), and the octanol/water partition coefficient (cLogP) (http://comptools.linc.ufpa.br/BChemRF-CPPred/) of wild type and mutated peptides were predicted.

In addition, the instability index (https://web.expasy.org/protparam/) and the hemolytic activity (https://webs.Iiitd.edu.in/Raghava/hemopi/batch.php) of peptides were calculated using the expasy server and the PROB score.

The tendency for β-sheet or α-helix aggregation of all peptides was anticipated using the Tango server (http://tango.Crg.Es/protected/academic/calculation.Jsp) [29].

Finally, the antigenicity (http://imed.med.ucm.es/Tools/antigenic.pl), allergenicity (https://www. ddg-pharmfac.net/AllerTOP), and toxicity (https://webs.iiitd.edu.in/raghava/toxinpred/design.php) of peptides were evaluated.

Then, MD has been carried out to study the biophysical properties of antimicrobial peptides [30].

The wild type peptide was positioned within a triclinic simulation box, maintaining a distance of 10 Å from the box edges, which contained the Spc216 water model. Molecular dynamics (MD) simulations were conducted utilizing the G43A1 force field [14,16,20,30]. To neutralize the overall charge of the peptides, Na^+^ and Cl^−^ ions were incorporated. Additionally, periodic boundary conditions were implemented for the hydrated protein system.

Following this setup, the systems underwent energy minimization through the steepest descent algorithm, succeeded by the conjugate gradients algorithm. Once energy minimization was completed, the system was equilibrated under constant volume (NVT) for 500 ps and constant pressure (NPT) for 1000 ps, during which the initial structural configuration remained fixed. Electrostatic interactions were calculated using the particle mesh Ewald (PME) method, with cut-off distances for electrostatic and van der Waals interactions set at 1 nm. The LINCS algorithm [31] was employed to constrain all covalent bonds involving hydrogen atoms during the production phase. Subsequently, a 200 ns MD simulation was performed under NPT conditions at 310 K with a time step of 0.001 ps for the wild type peptide [32]. The final structure of the wild type peptide in pure water was then utilized for MD simulations in boxes containing 30 %, 50 %, 70 %, and 100 % octanol, separately. The topology for the octanol molecule was generated using the prodrug server [33]. All input files of this study were upload into ZENODO repository (https://zenodo.org/records/14524367) to ensure reproducibility.

The dynamics of the peptides in an octanol medium are important because the existence of a polar head and a hydrocarbon chain can imitate the properties of lipid membranes [30,34]. In addition, because octanol has a simpler structure than cell membranes, it's easier to apply in an *in silico* environment. In pharmacology, the water-octanol partition coefficient is universally used to predict the pharmacokinetic properties of drug molecules, supplying a surprisingly good correlation with water-membrane partitioning [18]. The pure water and four octanol concentrations (30 %, 50 %, 70 %, and 100 %) can mimic the water, water-membrane interface, and membrane core environment. The octanol molecules were placed within the simulation box via the “gmx insert-molecules -nmol 200 -ci octanol.gro” command, which places octanol molecules randomly between water molecules. The number of octanol molecules can be changed using the “nmol” option to provide different concentrations of octanol inside the simulation box. The final structure of wild type RP9 in pure water was used to make all mutated structures using Swiss-PdbViewer software (https://spdbv.unil.ch/). The same MD procedures have been repeated for each mutated peptide. Then twenty MD (four peptides at five octanol concentrations), each one for 200 ns, were accomplished.

The number of water molecules, octanol, and ions in every octanol concentration was declared in Table 1.

**Table 1:** The number of waters, octanol molecules, and ions in different simulations in this work.

Wild type RP9 (RGSALTHLP)					
	Water	Octanol	Na^+^	Cl^−^	Total atom number
0 % Oct	1300	0	3	4	3992
30 % Oct	448	154	3	4	2976
50 % Oct	170	175	3	4	2352
70 % Oct	89	219	3	4	2549
100 % Oct	0	177	3	4	1862
LR-mut (RGSALTHLR)					
0 % Oct	1253	0	3	4	3861
30 % Oct	299	135	3	4	2349
50 % Oct	172	175	3	4	2368
70 % Oct	97	234	3	4	2733
100 % Oct	0	179	3	4	1892
KR-mut (RGSAKTHLR)					
0 % Oct	1079	0	–	3	4578
30 % Oct	289	135	–	3	2319
50 % Oct	197	197	–	3	2663
70 % Oct	129	314	–	3	3629
100 % Oct	0	182	–	3	1922
WP-mut (RGSAWTHLP)					
0 % Oct	1165	0	3	4	3599
30 % Oct	299	136	3	4	2361
50 % Oct	191	202	3	4	2697
70 % Oct	89	236	3	4	2731
100 % Oct	0	177	3	4	1874

The methodology for calculating the ΔG_sol_ of each peptide at different octanol concentrations comprised several steps. Initially, ten conformations were chosen from the final 50 ns of the molecular dynamics simulation for each peptide. Subsequently, “pqr” files were generated, and the free energy of solvation for each conformation was determined using the APBS server (https://server.poissonboltzmann.org/) [35]. Finally, average values were computed for each peptide across the various octanol concentrations. The simulation results were derived from 200 ns of molecular dynamics, analyzed using Gromacs commands. The mean and standard deviation of all parameters were then calculated for the last 50 ns of the simulation.

Also, the coordination number of water molecules around the NE atom of Arg1 of all peptides, which counts the water molecule number in the nearest neighboring distance (first peak of RDF), was calculated using the “gmx trjorder” command.

The percentage of residues that were within the secondary structures of peptides during the last 50 ns of MD was estimated via the “gmx dssp” command.

The “gmx covar” command was used for PCA analysis in this study, and the projections of trajectories onto the eigenvectors have been obtained via the “gmx anaeig” command [36].

The FEL (free energy landscape) of peptides in different octanol concentrations was calculated using the “gmx sham” command of the GROMACS package [37].

The analysis of frustration in energetic patterns of proteins, through the examination of single residue frustration or pairwise frustration contacts, can provide insights into the local stability, foldability, and functionality of proteins. Configurational frustration reflects the appropriateness of wild type interactions between two residues in comparison to alternative interactions that may occur in other compact structures. The frustration index quantifies the compatibility of a specific contact in relation to the entire range of potential contacts at that site, normalized by the variance of that distribution [38].

In the case of single residue frustration, if frustration indexes (FI) < −1, the interaction is highly frustrated. High local frustration often shows biologically important regions involved in the binding site or the catalytic site, or allostery and making clusters on the protein surface. If FI > 0.55 then the interaction is minimally frustrated.

In the case of configurational pairwise contact frustration, if FI < −1 then the interaction is highly frustrated, which means the energy contact residue pairs at that position are unfavorable for folding relative to other contact residues, and if FI > 0.78 then the interaction is minimally frustrated, which means the energy of contact residue pairs in that position is desirable for folding relative to other pairs and leads to strong stability [[38], [39], [40]].

In this study, we calculated the single residue frustration index and pairwise configurational frustration index of interacting pair residues of peptides for investigation of the stability of wild type and mutated peptides in different octanol concentrations. The final structures obtained from MD in all octanol concentrations were submitted to the frustratometer web server (http://frustratometer.qb.fcen.uba.ar) [38] for estimation of single residue frustration and pairwise configurational frustration indexes.

Additionally, due to the similarity of certain results, a one-way ANOVA analysis was conducted using IBM SPSS Statistics (Version 27) to assess the statistically significant mean parameters across various octanol concentrations. The p-values (significance values) indicating the differences between the means for each peptide were subsequently calculated.

### 2.2 Experimental methods

In the experimental section, aligning with the theoretical findings, the natural RP9 peptide and its KR-mut were procured from Shanghai Royobiotech Co., Ltd. (http://www.royobiotech.com/en/about.php). To assess the antimicrobial efficacy of these peptides, both gram-negative *Escherichia coli* (PTCC35218) and gram-positive *Staphylococcus aureus* (PTCC6538) were employed for the evaluation of the peptides' antimicrobial properties. A microbial assay was performed to determine the minimum inhibitory concentration (MIC) using a microplate microdilution method [41]. The bacterial strains were cultured overnight in a liquid LB medium at 37 °C. For inoculating the bacterial suspension into the 96-well microplate, the suspension was adjusted to a turbidity of 0.01 McFarland. The natural peptide and KR-mut were diluted with sterile distilled water, with sterile distilled water serving as the negative control and ampicillin as the positive control. Following the addition of an equal volume of bacterial suspension to the peptide solutions, the 96-well plate was incubated for a duration of 16–24 h at 37 °C. The MIC was defined as the range between the concentration of the last well exhibiting no bacterial growth and the subsequent lower concentration that permitted bacterial proliferation [42].

## 3 Results

### 3.1 Theoretical results

#### 3.1.1 Physicochemical properties of peptides

The physicochemical properties of antimicrobial peptides (AMPs) are crucial to their functionality; therefore, these properties have been examined through the analysis of peptide sequences (Table 2).

**Table 2:** Physicochemical results of peptides.

Peptide	MW	Octanol scale (kcal/mol)	Antigenicity	Amphipathicity	Total Charge	Dipole moment	Basic %	Allergenicity	GRAVY	Cell penetration% CP%	cLogP
Wild type RP9	951	1.92	1.044	0.43	+1	0.08	22.2	Non-allergen	−0.2	73.2	−4.87
LR-Mut	1010	3.59	1.023	0.71	+2	0.21	33.3	Non-allergen	−0.52	84.8	−6.11
KR-mut	1025	7.64	0.988	1.11	+3	0.47	44.4	Non-allergen	−1.38	88.1	−7.03
WP-mut	1024	1.08	1.005	0.43	+1	0.07	22.2	Allergen	−0.72	73.5	−4.19

The data presented in Table 2 indicates that the molecular weight (MW) of KR-mut is moderate, while its cell-penetrating ability surpasses that of other peptides, allowing for enhanced penetration into bacterial membranes.

The GRAVY parameter, which measures hydropathy, is lowest for KR-mut, suggesting a more hydrophilic nature due to its more negative GRAVY value.

This observation aligns with the reduced cLogP of KR-mut, further contributing to its hydrophilicity. Amphiphilicity, characterized by the dipole moment, plays a crucial role in the functionality of antibacterial peptides and is considered more significant than hydrophobicity in this context [43,44].

The dipole moment values for KR-mut are higher compared to other peptides, as shown in Table 2. This dipole moment facilitates the passive penetration of peptides through the lipid bilayer. Additionally, KR-mut exhibits a greater positive charge (+3e) and a more basic nature than its counterparts. The presence of positive and basic residues enhances the absorption of peptides into the negatively charged membranes of microorganisms, promoting electrostatic interactions with the phospholipid heads of the lipid bilayer, which may lead to improved antibacterial efficacy [9,28].

The propensity for aggregation into β-sheets or α-helices obtained from the sequence of peptides via Tango server among all peptides was negligible, indicating that none of the peptides exhibit a tendency to aggregate.

The instability index serves as a criterion for assessing the potential stability of a protein in a test tube environment. Proteins that are deemed stable typically have an instability index significantly below forty. For the wild type, LR-mut, and KR-mut peptides, the instability index was recorded at 20.86, while the WP-mut peptides exhibited a value of 41.6. Consequently, all peptides were determined to be stable.

In terms of hemolytic activity, all peptides displayed a PROB score of 0.49, which falls within a range of zero to one, where zero signifies nonhemolytic properties and one indicates hemolytic characteristics. Thus, all peptides demonstrated suitable hemolytic properties.

Furthermore, the antigenicity, allergenicity, and toxicity of the peptides were evaluated using the AllerTOP server. Notably, the antigenicity of the KR-mut peptide was found to be lower than that of the other peptides, and it exhibited neither allergenicity nor toxicity (Table 2).

In conclusion, following the analysis of the KR-mut sequence, the peptides RR-mut (RGSARTHLR) and WR-mut (RGSAWTHLR) were identified as potential candidates; however, they were ultimately disregarded due to their allergenic properties.

### 3.2 Molecular dynamics simulation results

Table 3 presents the time-averaged parameters recorded during the last 50 ns of 200 ns molecular dynamics simulation across various octanol concentrations. It includes the numerical average of these time-mean values for octanol concentrations ranging from 0 % to 100 %. The parameters analyzed include the structural drift or the backbone's root mean square deviation (RMSD), solvent accessible surface area (SASA), temperature, the radius of gyration (Rg), solvation free energy (ΔG_sol_), normalized potential energy of the peptides, the number of contacts (distances less than 6 Å) between peptides and water, as well as the number of hydrogen bonds formed between peptides and water (HBpep-sol), between peptides and octanol molecules (HBpep-oct), and within peptides themselves (HBpep-pep) for all peptides at each octanol concentration. Additionally, the numerical average of the backbone's root mean square fluctuation (RMSF) for the all residues of each peptide at each octanol concentration was also provided.

**Table 3:** The time-mean of results of the last 50 ns MD simulation of 200 ns MD simulation of peptides in each octanol concentrations and numerical average (the average of time-means in 0–100 % octanol concentrations) of parameters in the last column.

	Time-mean	0 % oct	30 % oct	50 % oct	70 % oct	100 % oct	Numerical Average
Wild type RP9	Temperature (K)	310.3 ± 4.8	309.7 ± 6.5	309.71 ± 6.8	309.87 ± 6.1	309.97 ± 6.5	–
	Normalized Potential (KJ/mol)	−14.3432	−8.956	−6.7264	−4.9922	−4.8502	−7.97 ± 3.92
	Rg (nm)	0.54 ± 0.03	0.56 ± 0.02	0.58 ± 0.03	0.61 ± 0.02	0.54 ± 0.01	0.57
	Backbone's Rmsd (nm)	0.17 ± 0.08	0.15 ± 0.01	0.19 ± 0.04	0.21 ± 0.04	0.260.01	0.20
	Sasa (nm^2^)	10.81 ± 0.65	11.26 ± 0.47	11.12 ± 0.54	11.62 ± 0.66	10.86 ± 0.38	11.13
	Average RMSF of residues (nm)	0.16 ± 0.03	0.12 ± 0.04	0.17 ± 0.08	0.18 ± 0.03	0.10 ± 0.02	0.15
	ΔG_sol_ (Kj/mol)	−949.88 ± 170	−879.07 ± 63	−933.8 ± 90	−972.1 ± 181	−854 ± 45	−917.8 ± 49.5
	Hbond pep-sol	23.83 ± 3.19	20.49 ± 2.98	19.01 ± 3.05	17.67 ± 3.96	–	20.25 ± 2.65
	Hbond pep-oct	–	0.44 ± 0.68	0.37 ± 0.65	0.49 ± 0.75	2.22 ± 1.39	0.88 ± 0.9
	Hbond pep-pep	2.43 ± 1.24	3.31 ± 1	2.57 ± 1.22	2.66 ± 1.51	2.52 ± 1.06	2.74 ± 0.39
	NC	531.31 ± 32	309.3 ± 27.8	210.96 ± 21.1	142.81 ± 19.2	–	298.6 ± 169.5
	Structure (%)	27	50	10	10	15	23 ± 17
	Random Coil (%)	53	47	64	66	49	56 ± 9
	Bend (%)	17	3	26	24	36	21 ± 12
LR-mut	Temperature (K)	309.98 ± 5.4	309.98 ± 7.3	310.18 ± 6.5	309.8 ± 6	310.11 ± 6.5	–
	Normalized Potential (KJ/mol)	−14.3901	−8.6006	−6.4287	−5.2658	−4.9599	−7.92 ± 3.88
	Rg (nm)	0.68 ± 0.11	0.53 ± 0.02	0.52 ± 0.01	0.53 ± 0.02	0.54 ± 0.01	0.56
	Backbone's Rmsd (nm)	0.31 ± 0.16	0.28 ± 0.02	0.09 ± 0.01	0.11 ± 0.03	0.23 ± 0.01	0.20
	Sasa (nm^2^)	12.85 ± 1.17	11.38 ± 0.52	11.05 ± 0.46	11.41 ± 0.5	10.68 ± 0.33	11.47
	Average RMSF of residues (nm)	0.38 ± 0.08	0.17 ± 0.03	0.11 ± 0.03	0.13 ± 0.03	0.1 ± 0.02	0.18
	ΔG_sol_ (Kj/mol)	−1174.6 ± 138	−1098.8 ± 43	−1002.4 ± 34	−1075.8 ± 58	−1024.4 ± 106	−1075.1 ± 67.6
	Hbond pep-sol	27.48 ± 3.76	18.86 ± 3.71	14.06 ± 2.81	13.71 ± 3.04	–	18.53 ± 6.4
	Hbond pep-oct	–	0.61 ± 0.81	0.77 ± 0.89	1.24 ± 1.1	3.84 ± 1.79	1.62 ± 1.51
	Hbond pep-pep	2.73 ± 1.49	6.97 ± 1.69	7.66 ± 1.53	7.42 ± 1.63	5.14 ± 1.47	6.19 ± 2.33
	NC	581.41 ± 45.9	272.01 ± 34.4	167.14 ± 20.3	146.31 ± 18.9	–	291.7 ± 200.8
	Structure (%)	0	34	34	34	20	24 ± 15
	Random Coil (%)	56	33	36	34	49	42 ± 10
	Bend (%)	44	33	30	32	31	34 ± 6
KR-mut	Temperature (K)	310.27 ± 4.8	309.95 ± 7.3	309.98 ± 6.4	309.93 ± 6.3	309.59 ± 6.3	–
	Normalized Potential (KJ/mol)	−14.0564	−7.8958	−5.7803	−4.5671	−4.1860	−7.30 ± 4.04
	Rg (nm)	0.61 ± 0.05	0.58 ± 0.02	0.59 ± 0.02	0.59 ± 0.03	0.52 ± 0.01	0.58
	Backbone's Rmsd (nm)	0.26 ± 0.07	0.21 ± 0.03	0.14 ± 0.06	0.13 ± 0.03	0.16 ± 0.01	0.18
	Sasa (nm^2^)	12.36 ± 0.82	12.25 ± 0.48	12.24 ± 0.46	12.03 ± 0.5	10.21 ± 0.3	11.82
	Average RMSF of residues (nm)	0.21 ± 0.07	0.13 ± 0.03	0.15 ± 0.01	0.17 ± 0.02	0.06 ± 0.02	0.15
	ΔG_sol_ (Kj/mol)	−719.4 ± 70	−1560.4 ± 48	−1515.1 ± 78	−1741.35 ± 62	−1678.3 ± 53	−1442.9 ± 414.4
	Hbond pep-sol	30.47 ± 3.7	20.60 ± 3.35	15.41 ± 2.74	18.04 ± 3.27	–	21.13 ± 6.57
	Hbond pep-oct	–	0.8 ± 0.9	0.94 ± 0.98	0.77 ± 0.89	2.05 ± 1.35	1.14 ± 0.61
	Hbond pep-pep	2.75 ± 1.45	5.50 ± 1.73	6.84 ± 1.55	6.57 ± 1.51	6.86 ± 1.3	5.42 ± 1.78
	NC	638.14 ± 53	296.09 ± 27.3	180.81 ± 11	218.14 ± 24.5	–	333.3 ± 208.8
	Structure (%)	56	26	38	44	8	34 ± 18
	Random Coil (%)	44	50	48	44	57	49 ± 5
	Bend (%)	0	24	14	11	34	17 ± 13
WP-mut	Temperature (K)	310 ± 5.5	309.64 ± 7.3	310.08 ± 6.3	309.91 ± 5.8	310.24 ± 6.6	–
	Normalized Potential (KJ/mol)	−14.372	−8.4697	−6.4712	−5.2638	−4.7797	−7.87 ± 3.9
	Rg (nm)	0.56 ± 0.03	0.54 ± 0.02	0.52 ± 0.01	0.59 ± 0.06	0.52 ± 0.01	0.55
	Backbone's Rmsd (nm)	0.19 ± 0.11	0.13 ± 0.01	0.25 ± 0.03	0.25 ± 0.06	0.13 ± 0.01	0.19
	Sasa (nm^2^)	11.06 ± 0.71	10.9 ± 0.64	10.55 ± 0.42	11.73 ± 1.04	9.93 ± 0.31	10.83
	Average RMSF of residues (nm)	0.32 ± 0.09	0.12 ± 0.05	0.12 ± 0.02	0.21 ± 0.06	0.08 ± 0.02	0.18
	ΔG_sol_ (Kj/mol)	−719.4 ± 70	−678.29 ± 67	−623.6 ± 49	−874.3 ± 117	−691.64 ± 38	−717.4 ± 94.4
	Hbond pep-sol	20.80 ± 3.56	16.66 ± 2.64	13.70 ± 4.28	16.46 ± 4.08	–	16.90 ± 2.92
	Hbond pep-oct	–	0.42 ± 0.67	0.4 ± 0.66	0.67 ± 0.84	1.9 ± 1.3	0.85 ± 0.71
	Hbond pep-pep	4.15 ± 1.58	4.46 ± 1.18	5.73 ± 2.33	3.43 ± 1.94	4.44 ± 0.96	4.44 ± 0.96
	NC	532.76 ± 32.8	255.51 ± 31.4	171.22 ± 17.9	145.8 ± 23	–	276.3 ± 177.2
	Structure (%)	56	18	6	6	11	19 ± 21
	Random Coil (%)	44	49	55	64	67	56 ± 10
	Bend (%)	0	31	40	31	22	25 ± 15

NC: Number of contacts between peptide and water molecules, Structure = β-bridge + turn.

The time-mean values and the small standard deviations of temperature, RMSD, Rg, SASA, and RMSF for peptides at various octanol concentrations indicated that all peptides achieved a plateau and reached thermal and structural equilibrium during the last 50 ns of the molecular dynamics simulation.

The distribution of all parameters was found to be normal, and an F-test for homogeneity of variance, specifically Levene's test, was conducted. The P-values obtained from the F-test for each parameter pair across all concentrations were zero, indicating that the variances were not homogeneous. Consequently, the Games-Howell post hoc analysis was employed for pairwise comparisons of the parameters. According to the results of the Games-Howell analysis, all parameters across all concentrations exhibited significant differences at the 0.05 level [45], except some certain variables that had P-values exceeding 0.05, indicating no significant difference.

These values had red color in Table 3. To facilitate the comparison of these parameters, one of these variables was disregarded, and only the most impactful parameters that demonstrated significant differences were compared.

The trendline slope of the time-mean of parameters (over the last 50 ns of MD) versus octanol concentration (after omission of one of the insignificant time-means) and coefficient of determination (R^2^) in this plot were indicated in Table 4.

**Table 4:** The trendline slope and coefficient of determination (R^2^) of plot time-mean of parameters versus octanol concentration.

	Wild type		LR-mut		KR-mut		WP-mut	
	slope	R^2^	slope	R^2^	slope	R^2^	slope	R^2^
RMSD	0.002	0.97	−0.003	0.79	−0.002	0.94	0.001	0.4
Rg	0.001	0.92	−0.001	0.65	−0.001	0.89	−0.0004	0.84
SASA	0.01	0.79	−0.02	0.84	−0.03	0.78	−0.007	0.15
RMSF	−0.0005	0.34	−0.003	0.78	−0.001	0.91	−0.002	0.71
Hbondpep-sol	−0.09	0.98	−0.2	0.92	−0.19	0.78	−0.07	0.57
Hbondpep-oct	0.025	0.72	0.046	0.84	0.016	0.67	0.021	0.81
NC	−5.6	0.97	−6.35	0.89	−6.28	0.8	−5.61	0.89
Normalized potential energy (kj/mol)	0.09	0.85	0.09	0.82	0.09	0.82	0.09	0.83
Percentage of residue in quasi-secondary structure	−0.033	0.51	−0.04	0.6	−0.1	0.75	−0.16	0.78
Percentage of residues in random coil	0.05	0.64	0.04	0.63	0.03	0.85	0.06	0.98

The trendline of the RMSD plot exhibited a decreasing trend (negative slope) for both LR-mut and KR-mut, while an increasing trend (positive slope) was observed for the wild type and WP-mut peptides (Table 4). Notably, the KR-mut demonstrated a more continuous decline as octanol concentration rose (Table 3) compared to the LR-mut. Furthermore, the numerical average of the RMSD value for KR-mut across all octanol concentrations was lower than that of the other peptides (Table 3).

The radius of gyration (Rg) of a protein pertains to the spatial distribution of atoms relative to its center of mass. The solvent accessible surface area (SASA) plays a vital role in examining conformational alterations within protein structures. This metric is particularly important for analyzing the interactions between the peptide and the surrounding solvent. The trendline slope of time-mean versus octanol concentration for both Rg and SASA (after excluding one insignificant value) was positive for the wild type form and negative for the mutations. The slopes of the trendlines for Rg in both LR-mut and KR-mut were comparable (Table 4). In various peptides, the time-mean of Rg values shows variability without a constant pattern in response to increasing octanol concentrations; however, for the KR-mut, a steady decline in time-mean was noted (Table 3).

The trendlines for KR-mut in the SASA plot exhibited more negative slope compared to other peptides.

The root mean square fluctuation (RMSF) per residue of the peptide backbone illustrates the movement of each residue from its original position over the final 50 ns of the MD simulation. A higher RMSF in a specific region of the protein, excluding the C- and N-terminal ends, suggests that this area behaves as a random coil or loop. Fig. S1 presents the RMSF per residue for each peptide across all concentrations. Notably, the RMSF values for the C-terminal residues of the peptides were lower than those for the N-terminal residues across all octanol concentrations.

**Fig. 1:**
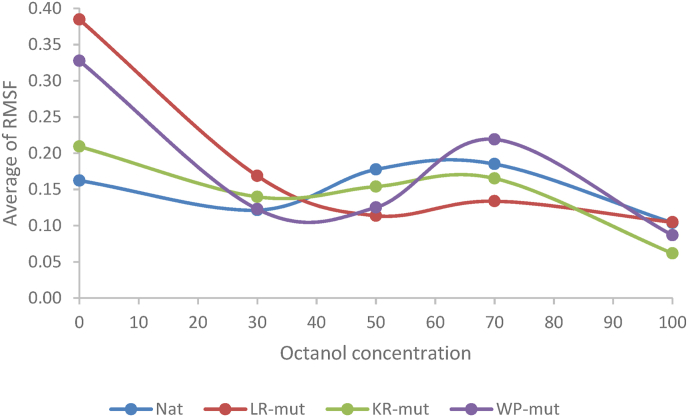
The average RMSF of residues in each peptide versus different concentrations of octanol.

Fig. 1 displays the average RMSF of the backbone residues for each peptide in relation to varying octanol concentrations.

The information illustrated in Fig. 1 reveals that an overall increase in octanol concentration leads to a decrease in the backbone's RMSF for all peptides, with a constant negative trendline slope. Notably, the slope for LR-mut was more negative, while KR-mut displayed a more robust linear regression with respect to octanol concentration (R^2^ = 0.9) when compared to the other peptides, as detailed in Table 4.

Additionally, the average of RMSF values at all octanol concentrations for both KR-mut and wild type peptides were found to be lower than those of the other peptides, as shown in Table 3. This indicates that KR-mut exhibited a steadier behavior within the membrane environment.

In contrast, the flexibility, or RMSF, of the C-terminal residues of the peptides was observed to be lower than that of the N-terminal across all octanol concentrations, as depicted in Fig. S1. This observation can be explained by the presence of threonine at position 7, which is located near the C-terminal.

The time-mean and the numerical average of the number of intramolecular hydrogen bonds within peptides (Hbpep-pep) are presented in Table 3. This parameter remained relatively constant, in the wild type peptide with changes in octanol concentration. In contrast, all mutations displayed a nearly significant difference in this parameter, which varied with octanol concentration (Table 3). Both KR-mut and LR-mut demonstrated a higher number of intramolecular hydrogen bonds compared to other peptides, with KR-mut exhibiting a more pronounced positive slope in its trendline.

Given that the differences in the time-mean of hydrogen bonds between peptides and water molecules (Hbond pep-sol) across various octanol concentrations were significant. The trendline slope of time-mean versus octanol concentration for all peptides was negative, indicating a decrease in this parameter with increasing octanol concentration; however, the slope was more negative for both LR-mut and KR-mut (Table 4). Notably, KR-mut had a higher numerical average of intermolecular hydrogen bonds with water molecules than all other peptides, contributing to improved solubility (Table 3).

The trendline slope representing the time-mean count of hydrogen bonds formed between peptides and octanol molecules across varying concentrations (Hbond pep-oct) was found to be positive. This parameter exhibited an increase with rising octanol concentrations, with LR-mut demonstrating the steepest positive slope. Furthermore, LR-mut also recorded the highest average number of hydrogen bonds with octanol, suggesting that it has superior hydrogen bonding interactions compared to the other peptides. Conversely, the standard deviation of this parameter was notably high across all peptides, indicating significant variability; however, KR-mut displayed a lower standard deviation, implying a more uniform interaction with octanol or membrane components (Table 3, Table 4).

In contrast, the slope of the trendline for the time-mean of the number of contacts between peptides and water molecules (NC) was negative, showing a decrease as octanol concentration increased from 0 % to 70 % (with no water present at 100 % octanol) for all peptides. Both LR-mut and KR-mut exhibited steeper negative slopes than the other peptides (Table 4). This trend was understandable, as the number of water molecules decreases at higher octanol concentrations.

Importantly, KR-mut demonstrated the highest numerical average number of peptide-water contacts across all concentrations when compared to the other peptides, which may enhance its solubility and antibacterial properties (Table 3).

The free energy of solvation (ΔG_sol_) was derived from conformations obtained through molecular dynamics simulations using APBS software. The numerical average of ΔG_sol_ across all octanol concentrations was more negative for KR-mut, indicating improved solubility for this variant (Table 3).

The time-averaged potential energy of all systems was calculated over the last 50 ns of molecular dynamics simulations. To evaluate the normalized potential energy of the peptides, the potential energy was divided by the total number of atoms (Table 1). The trendline slope of the time-averaged normalized potential energy plotted against octanol concentration for each peptide was positive, indicating a reduction in system stability with increasing octanol concentration (Table 4). The comparable slopes of the time-averaged values (Table 4) and the similar numerical averages across all peptides (Table 3) suggest that mutations did not significantly influence the rate of instability induced by octanol increase.

Given the short length of the peptides, they displayed a minimal presence of secondary structure. Throughout the molecular dynamics simulations, none of the peptide residues contributed to the formation of an α-helical structure.

The trendline slope representing the time-mean of the percentage of residues contributing to quasi-structured conformations (β-bridge plus turn) exhibited a negative trend for all peptides, indicating a decline and disruption in these conformations as octanol concentration increased. Conversely, the trendline for the time-mean percentage of residues associated with random coil and bend structures showed an upward trend, signifying an increase in random coil conformations with rising octanol levels. It is noteworthy that the KR-mut peptide displayed the lowest slope, implying that it experienced a more gradual loss of secondary structure compared to the other peptides in response to elevated octanol concentrations (Table 4).

Among the peptides studied, KR-mut exhibited the highest numerical average of quasi-structured elements (comprising β-bridges and turns), while simultaneously showing the lowest percentage of bends and nearly the least numerical average of random coil structures across all octanol concentrations when compared to the other peptides (Table 3). This indicates that KR-mut possesses a greater degree of quasi-secondary structure than its counterparts.

Fig. S2 presents the radial distribution function (RDF) plot, which illustrates the spatial arrangement of water's oxygen atoms to the nitrogen atom of Arg1 across all peptides at octanol concentrations ranging from 0 % to 70 %. Table 5 details the height of the first peak in the RDF plot, as well as the distance from the oxygen atoms of water to Arg1 at this peak. It is noted that the height of the first peak in the RDF plot increases with higher octanol concentrations, suggesting an improved capacity for water absorption by the peptides at elevated octanol levels. As a result, the interaction between the peptides and water becomes more pronounced at increased octanol concentrations. The distance of water to Arg1 at the first peak of the RDF plot remained constant across all peptides, indicating that all peptides engaged with water molecules at a distance of approximately 3 Å at first this peak of RDF.

**Table 5:** The distance between the nitrogen atom of the Arg 1 side chain and the oxygen atoms of the surrounding water, as well as the height of the first peak in the radial distribution function (RDF) plot (HR) and the coordination number for all peptides across various octanol concentrations and numerical average (NA) of these parameters.

		0 %	30 %	50 %	70 %	NA
Native RP9	Distance (nm)	0.3	0.29	0.29	0.28	0.29 ± 0.01
	HR	0.66	2.38	5.38	15.4	5.95 ± 6.59
	Coordination number	0.46 ± 0.52	0.43 ± 0.51	0.29 ± 0.46	0.30 ± 0.46	0.37 ± 0.09
LR-mut	Distance (nm)	0.29	0.29	0.29	0.28	0.29 ± 0
	HR	0.63	2.97	5.54	11.81	5.24 ± 4.82
	Coordination number	0.56 ± 0.54	0.31 ± 0.47	0.38 ± 0.50	0.23 ± 0.42	0.35 ± 0.1
KR-mut	Distance (nm)	0.29	0.28	0.29	0.28	0.29 ± 0
	HR	0.68	3.13	6.14	13.57	5.88 ± 5.59
	Coordination number	0.36 ± 0.49	0.28 ± 0.45	0.32 ± 0.47	0.64 ± 0.52	0.4 ± 0.16
WP-mut	Distance (nm)	0.29	0.29	0.29	0.29	0.29 ± 0
	HR	0.62	2.93	5.39	15.31	6.06 ± 6.46
	Coordination number	0.34 ± 0.48	0.37 ± 0.49	0.28 ± 0.45	0.35 ± 0.48	0.33 ± 0.04

Additionally, the RDF plot facilitated the determination of the coordination number of nitrogen atoms surrounding Arg1. A comprehensive analysis of the variations in the coordination number of water molecules during the octanol growth process indicated a reduction in this metric for both wild type and LR-mut peptides, while it remained unchanged for WP-mut. In contrast, an increase was observed in KR-mut. Furthermore, the numerical average coordination number of water molecules in KR-mut was found to exceed that of the other peptides, as detailed in Table 5.

The potential energy surface of proteins, also referred to as the phase space of proteins, is characterized by its complexity and enormous, illustrating the probability of various protein conformations corresponding to specific bond lengths, angles, and torsion angles. Principal component analysis (PCA) serves as a mathematical method for reducing dimensions, relying on the covariance matrix derived from the movements of Cα atoms in proteins. The eigenvectors obtained from this analysis provide guidance within the conformational space, representing the collective movements of atoms along designated directions. Meanwhile, the eigenvalues indicate the mean square fluctuations of atoms along corresponding eigenvectors, reflecting the variance explained by each principal component (PC). Through this analytical approach, it is possible to ascertain the original motion vectors of a protein within its phase space. Research has demonstrated that the majority of a protein's internal movements are confined to a subspace of significantly reduced dimensions [37].

**Fig. 2:**
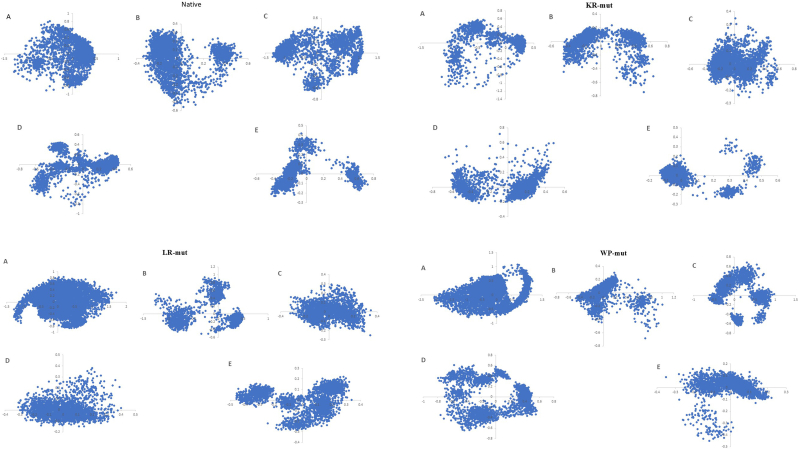
The scatter plot of PC1 versus PC2 of Cα displacement of peptides in different concentrations (A: 0 %, B: 30 %, C: 50 %, D: 70 %, E: 100 % octanol concentration) during 200 ns MD.

In this study, PCA analysis was performed to evaluate the Cα displacement of peptides at different octanol concentrations throughout 200 ns in molecular dynamics simulations. A scree plot was created for each peptide across all octanol concentrations, ranking the eigenvalues from highest to lowest. The findings revealed that all peptides displayed a noticeable bend in the scree plot after the second principal component (PC2), leading to a subsequent linear trend. As a result, only the first two principal components (PC1 and PC2), which had the highest eigenvalues, were selected for the PCA analysis. Table S1 provides the first and second eigenvalues obtained from the PCA analysis of peptides at various octanol concentrations, while the other eigenvalues were deemed insignificant. Fig. 2 depicts the scatter plot of PC1 versus PC2 concerning the Cα displacement of peptides across different octanol concentrations during the 200 ns molecular dynamics simulation.

As demonstrated in Fig. 2, the conformational variability of peptides in aqueous environments is significant; however, an increase in octanol concentration leads to a restriction of peptide conformations to a more limited range. This phenomenon is consistent with the observed decrease in root mean square fluctuation (RMSF) at elevated octanol levels.

The free energy landscape (FEL) offers valuable insights into the folding pathways of peptides, which are influenced by the primary components PC1 and PC2, along with corresponding structural changes. Within this context, the free energy is intrinsically linked to the probability density function that quantifies the likelihood of a conformation occurring at specific values of PC1 and PC2 [36,37], as illustrated in Fig. S3. The red valleys depicted in Fig. S3 signify low-energy regions or thermodynamically stable states within the peptide phase space at different octanol concentrations. Conversely, the expansive blue areas represent high free energy states, indicating lower probabilities of occurrence. The narrowness of the red regions across all states shown in Fig. S3 implies a limited number of stable, low-energy conformations.

Additionally, we concatenated the trajectories of each peptide across all octanol concentrations, resulting in a 1000 ns single trajectory for each peptide. Given that all trajectories at varying octanol concentrations pertain to the same peptide, we were able to concatenate these trajectories. Following this, principal component analysis (PCA) was performed on the Cα movements within the concatenated trajectories. The analysis of the PC1-PC2 plots, along with their corresponding free energy landscapes, revealed that the KR-mut peptide displayed more restricted conformations in comparison to the other peptides (Fig. S4).

Local energetic frustration evaluates the efficiency of interactions in promoting folding by contrasting its energy with the random interactions that might occur within the protein chain in non-native states. Findings from the frustratometer web server, based on the final structures, demonstrate that in pure water or at 0 % octanol concentration, neither the KR-mut nor the WP-mut peptides exhibited any highly frustrated single residues. In contrast, the wild type and LR-mut peptides showed 1 and 2 highly frustrated single residues, respectively (Fig. S5). This finding emphasizes the enhanced stability of KR-mut relative to the other peptides in pure water.

Table 6 presents the numerical averages of counts for high and minimally frustrated single residues, as well as residue-residue contacts across all octanol concentrations for the final structures of the peptides derived from molecular dynamics simulations.

**Table 6:** The numerical average of counts of high and minimally frustrated (HF and MF) single residue and residue–residue contacts (HF-pair and MF-pair) across all octanol concentrations in the final structures derived from MD.

	HF	MF	HF-pair	MF-pair
Wild type	1.2	2.6	0.8	0.34
KR-mut	1.6	3.8	1.4	6
LR-mut	2	3.4	2.2	5.2
WP-mut	1.4	2.4	1.4	4.4

The numerical average of the number of highly frustrated pairwise interactions across all concentrations of wild type, KR-mut, and WP-mut peptides, was found to be low. Notably, the KR-mut peptide exhibited a higher average of minimally frustrated pairwise interactions compared to the other peptides across all concentrations. This observation implies that KR-mut possesses enhanced stability throughout all octanol concentrations.

Moreover, the analysis reveals that an increase in octanol concentration typically results in a higher number of residues exhibiting highly frustrated pairwise interactions, which may lead to increased instability of the peptides at higher octanol levels.

In the final structures obtained from molecular dynamics simulations (MD), the side chains of arginine (Arg) and lysine (Lys) residues in all peptides extend beyond the peptide backbone. This extension facilitates stronger electrostatic interactions between the peptides and the cell membrane, enabling the rotation and insertion of the hydrophobic side chains of Arg or Lys into the membrane (Fig. S6).

Additionally, a visual assessment of the final peptide structures across all concentrations indicated that, in octanol solution, water and octanol molecules tended to aggregate. This behavior can be attributed to the hydrophobic interactions among the octanol molecules.

Overall, the physicochemical properties and MD findings suggest that KR-mut is likely to exhibit greater stability and antibacterial efficacy. Consequently, KR-mut was selected for the experimental antibacterial evaluation.

## 4 Experimental results

The antibacterial efficacy of the wild type RP9 and KR-mut peptides is presented in Table 7. The minimum inhibitory concentration (MIC) of the mutant peptide against the gram-positive bacterium *Staphylococcus aureus* was determined to be 363.5 μg ml^−1^, while for the gram-negative bacterium *Escherichia coli*, it was 500 μg ml^−1^. The findings indicate that the KR-mut peptide exhibits significantly stronger antimicrobial activity against both gram-positive and gram-negative bacteria compared to the wild type peptide. Furthermore, a comparative analysis of the antibacterial properties of the mutant peptide revealed a greater inhibitory effect on gram-positive bacteria than on gram-negative bacteria.

**Table 7:** MIC results of peptides.

Peptide	MIC (μg/ml) of *S. aureus*	MIC (μg/ml) of *E. coli*
Wild type RP9	727>	>500
KR mutant	363.5	500
Negative control	/	/
Positive control	3.90	15.62

MIC: Minimal inhibitory concentration of the antimicrobial peptide.

Positive control: Ampicillin.

Negative control: Distilled water.

## 5 Discussion

Antimicrobial peptides (AMPs) represent a novel category of antimicrobial agents that possess significant potential for developing new antibiotics to address the pressing issue of antibiotic-resistant microorganisms worldwide. These peptides have been discovered in a variety of species, where they function to counteract pathogenic microorganisms. The interaction and incorporation of AMPs into the cell membrane result in disruption of the membrane integrity, ultimately causing cell death [15].

The water-octanol system serves as an effective biomimetic model for the water-membrane interface. Additionally, the relatively simple structure of octanol compared to the membrane enhances its applicability in MD, thereby accelerating the MD process. The amphiphilic characteristics of octanol facilitate a diverse range of interactions with particular functional groups [18].

Biophysical studies that investigate the incorporation of amino acids or short polypeptides into "membrane-mimicking" environments, particularly n-octanol, have typically been employed to estimate the likelihood of amino acids entering the membrane core [46]. Peptide RP9, which is derived from the white blood cells of crocodiles, exhibits both antimicrobial and anticancer activities [11]. The development of antibacterial peptides requires careful consideration of several essential factors, such as positive charge, basic residues, amphipathic characteristics, and solubility. To improve positive charge and hydrophobicity, the peptides KR-mut (RGSAKTHLR), LR-mut (RGSALTHLR), and WP-mut (RGSAWTHLP) were designed.

The physicochemical properties of peptides were initially assessed through bioinformatics servers. Among the wild type peptides and their various mutations, the KR-mut variant displayed a greater positive charge, enhanced hydrophilicity, and improved amphiphilicity, which aid in its ability to penetrate cell membranes. Moreover, KR-mut showed no potential for allergenicity and presented lower levels of antigenicity, instability, and hemolytic activity when compared to other mutations and wild type peptides. All peptides were also determined to be non-toxic. As a result, the biophysical attributes of KR-mut were found to be superior to those of the other peptides. Biswal and colleagues noted that the density, volume, and solvent-accessible surface area (SASA) obtained from simulations in an octanol environment play a significant role in the efficacy of synthetic cationic polypeptides [22]. Following this, the changes in these parameters during molecular dynamics simulations of the mutations in pure water and at four different octanol concentrations (30, 50, 70, and 100 %) were thoroughly examined.

The results from the twenty molecular dynamics simulations of peptides in water, along with four different concentrations of octanol, revealed that all systems attained stable configurations. Only minor variations were noted in the thermodynamic properties at each concentration. The low standard deviations in temperature and the root mean square deviation (RMSD) of the peptides during the final 50 ns of all simulations suggested that the simulation duration was adequate.

The antibacterial effectiveness of peptides is linked to their ability to penetrate the cell membrane. An analysis of the trendline of the variation of the backbone's RMSD versus octanol concentration indicated that, for KR-mut and LR-mut, there was a continuous decrease in RMSD with increasing octanol concentration, unlike the other peptides (Table 4). This observation implies that KR-mut and LR-mut possess a more stable structure, exhibiting fewer structural fluctuations within the cell membrane. Furthermore, the lower numerical average of the RMSD value for KR-mut compared to the other peptides suggests reduced atomic movement at elevated octanol concentrations or within the membrane, which may enhance its antibacterial efficacy.

The negative slope observed in the trendline for Rg and SASA of the mutations indicates a decline in these parameters as octanol concentration increases. Notably, the more pronounced negative slope of KR-mut in the SASA trendline (Table 4) implies a greater rate of shrinkage upon entering the membrane, facilitating improved penetration of KR-mut into the microbial membrane and thereby augmenting its antibacterial properties. The reductions in Rg and SASA for KR-mut at 100 % octanol concentration align with the observed decrease in RMSD at this concentration.

The decrease in SASA for both linear and cyclic peptide forms in octanol solvent, as reported by Sabuti et al. [47], corroborates our findings.

A similar trend was noted concerning the average RMSF of residues, indicating that the numerical average of RMSF values across all octanol concentrations for both KR-mut and wild type peptides was lower than that of the other peptides (Table 3). This decrease in flexibility and disorder, coupled with the uniform behavior of KR-mut in relation to rising octanol concentrations, may promote its ability to penetrate membranes and improve its antibacterial efficacy.

Also, the steric hindrance introduced by the hydroxyl group of the threonine side chain reduces the flexibility of the C-terminal, which is consistent with the observations made by Sabuti et al. [47].

A general decline in the radius of gyration (Rg), solvent accessible surface area (SASA), and the average numerical value of RMSF for the peptides across all concentrations can enhance membrane penetration. Conversely, the KR-mut peptide demonstrated a more pronounced increase in the numerical average of the number of intramolecular hydrogen bonds relative to the other peptides. Research conducted by Frazee et al. [48] has shown that octanol solvent notably facilitates the formation of intramolecular hydrogen bonds, which is consistent with our observations.

A comparable trend was observed in the numerical average of intermolecular hydrogen bonds formed between KR-mut and water molecules. These findings imply an enhancement in both stability and solubility for this peptide. The standard deviation of the numerical average hydrogen bonds between KR-mut and octanol molecules was lower than that of other peptides, indicating a more uniform interaction of KR-mut with octanol or membrane components.

Moreover, KR-mut exhibited a higher numerical average of the number of peptide-water contacts at all concentrations when compared to the other peptides, contributing to its superior solubility. Additionally, the more negative average value of ΔG_sol_ across all octanol concentrations for KR-mut aligns well with the intermolecular hydrogen bonds, the number of contacts, and various biophysical properties (including the GRAVY scale, octanol scale, and cLogP) of this peptide. This suggests enhanced hydrophilicity, solubility, and antibacterial efficacy relative to other peptides. Chen et al. have highlighted that solubility in aqueous environments is a vital characteristic of antibacterial peptides [49].

As the concentration of octanol increases, the normalized potential energy correspondingly rises, resulting in a reduction of peptide stability at higher octanol concentrations. The observed decline in the quasi-structured conformations of the peptides with increasing octanol aligns with established knowledge regarding the impact of alcohols on protein denaturation. Furthermore, the relatively gentle positive slope of the graph illustrating random coil structures in relation to octanol concentration for KR-mut, in contrast to other peptides, indicates that KR-mut preserve its secondary structure in an octanol-rich environment.

The RDF analysis of the oxygen atoms in water relative to the nitrogen atom of Arg1 reveals an increased ability of peptides to retain water when octanol is present (Table 5). This finding is consistent with the research conducted by Sabuti et al. [47], which demonstrated that the interaction strength between linear peptides and water is enhanced in octanol solutions compared to pure water.

In contrast, the decrease in the coordination number observed for both wild type and LR-mut peptides, while remaining unchanged for WP-mut, can be attributed to a reduced number of water molecules at higher octanol concentrations. This aligns with the findings of Mortazavi et al. [14], who noted a reduction in the coordination number surrounding the nitrogen atom of lysine as octanol concentration increased. Similarly, Jahanbin et al. reported a similar decline in the coordination number of Cγ in peptides linked to Alzheimer's disease with rising alcohol concentrations [50].

Notably, contrary to other peptides and our initial expectation, the coordination number for KR-mut rises as octanol concentrations increase (Table 5). This rise could potentially improve the solubility of this peptide in high octanol environments. Additionally, the elevated numerical average coordination number for KR-mut suggests enhanced water absorption at all octanol concentrations, which aligns with a greater formation of hydrogen bonds between this peptide and water molecules, supporting earlier research on KR-mut.

The PC1-PC2 plots of the peptides during molecular dynamics simulations at different octanol concentrations demonstrated that octanol constrained the conformation and flexibility of the peptides, which corresponds with the observed decrease in root mean square fluctuation (RMSF) after octanol was introduced. Notably, the KR-mut peptide exhibited fewer conformational states than the other peptides, particularly at 100 % octanol concentration, which mimics a membrane-like environment. Frazee's research indicates that octanol can elicit distinct conformational behaviors [48], which aligns with our findings.

Additionally, the free energy landscape (FEL) analysis of the peptides suggests that at higher octanol concentrations, the conformations of the peptides are confined to narrow low-energy regions, leading to a limited array of stable conformations. The FEL plots (Fig. S3) corroborated the PC1-PC2 plots of the peptides (Fig. 2). These observations were further substantiated through principal component analysis (PCA) conducted on the combined trajectories of each peptide across various octanol concentrations (Fig. S5).

The average counts of highly frustrated and minimally frustrated single residues, as well as pairwise contacts across all octanol concentrations, indicated that KR-mut had fewer highly frustrated residues and a greater number of minimally frustrated single residues, along with superior pairwise contacts compared to the other peptides, suggesting a more stable structural configuration.

Theoretical predictions imply that LR-mut may demonstrate appropriate antibacterial properties following KR-mut.

The theoretical findings ultimately indicated that KR-mut is expected to demonstrate greater bactericidal efficacy than both the wild type peptide and other mutations. To confirm this hypothesis, both KR-mut and the wild type peptides were obtained, and their antimicrobial properties were evaluated through a minimum inhibitory concentration (MIC) comparison test.

In this assessment, *Escherichia coli* and *Staphylococcus aureus* were selected as representatives of gram-negative and gram-positive bacteria, respectively, with the former characterized by a double-membrane cell wall and the latter by a single cell membrane. The experimental outcomes revealed that KR-mut exhibits enhanced antimicrobial properties in comparison to the natural RP9 peptide against both types of bacteria, thereby supporting the theoretical predictions. Notably, its antimicrobial activity was particularly pronounced against the gram-positive *Staphylococcus aureus*. Yeaman et al. observed that antimicrobial peptides target the lipopolysaccharide found in the membranes of gram-negative bacteria [51]; however, structural modifications, such as the incorporation of sugars and peptides, may reduce their lethal efficacy against these organisms. In contrast, teichoic acid, which is present in gram-positive bacteria, experiences fewer modifications than lipopolysaccharides. The structure of teichoic acid in *S. aureus* typically comprises approximately 24 glycerophosphate repeating units, each contributing one negative charge from the phosphate group [52]. Consequently, the antimicrobial effects of KR-mut, which possesses three positive charges, are more effective against gram-positive microorganisms containing teichoic acid than those of the wild type RP9 peptide, which has only one positive charge.

The biophysical properties obtained from molecular dynamics simulations were consistent with the experimental results; however, the molecular dynamics simulations of peptides in a water-octanol environment did not reveal significant differences between gram-positive and gram-negative bacteria. Nonetheless, these factors may affect the enhancement or reduction of bactericidal properties as a result of peptide mutations.

In this investigation, the findings from the minimum inhibitory concentration (MIC) tests supported the bioinformatics analysis, indicating that the KR-mut peptide (RGSAKTHLR) demonstrates enhanced antibacterial efficacy compared to the wild type RP9 peptide. This peptide shows stability and solubility while presenting a low risk of immunogenicity, making it a viable candidate for the development of antibiotic peptides. It holds considerable promise for the progression of peptide-based antibiotics.

Additionally, this novel peptide has the potential to be a significant asset in the advancement of future therapeutic approaches aimed at antibacterial effectiveness. The research conducted by Torrent et al. demonstrated that molecular dynamics simulations of peptides within a water-octanol system, combined with bioinformatics analysis, can provide an effective and swift model for comprehending the characteristics and dynamics of intricate bacterial cell envelopes, as well as for the development of antibacterial peptides [53,54]. The computational techniques utilized in this study to evaluate antimicrobial peptides offer a valuable and economical strategy that enhances the accurate prediction and rational design of new candidate antibiotic peptides.

## CRediT authorship contribution statement

**Mahya Anahid:** Writing – original draft, Methodology, Data curation. **Karim Mahnam:** Writing – review & editing, Validation, Supervision, Project administration, Data curation. **Behnaz Saffar:** Visualization, Validation, Methodology, Data curation, Conceptualization.

## Future suggestion

It is recommended that other mutations, specifically LR-mut and WP-mut peptides, be synthesized and their antibacterial properties compared with those of the wild type RP9 peptide through experimental methods.

Additionally, the potential of the mean force (PMF) for both wild type and KR-mut peptides during their passage through the cell membranes of *E. coli* and *S. aureus* should be evaluated using the umbrella sampling technique. This investigation may provide insights into the theoretical foundations for the enhanced effectiveness of KR-mut on the *S. aureus* membrane.

Moreover, subsequent experimental studies should prioritize the exploration of the anticancer properties associated with this peptide.

## Funding statement

No funding was received in this study.

## Declaration of competing interest

The authors declare that they have no known competing financial interests or personal relationships that could have appeared to influence the work reported in this paper.

## Data Availability

Data will be made available on request.

## References

[1] Yount N.Y., Yeaman M.R. (2004). Multidimensional signatures in antimicrobial peptides. Proc. Natl. Acad. Sci. U.S.A..

[2] Cherkasov A., Jankovic B. (2004). Application of ‘inductive’ QSAR descriptors for quantification of antibacterial activity of cationic polypeptides. Molecules.

[3] Escribá P.V., Busquets X., Inokuchi J.I., Balogh G., Török Z., Horváth I., Harwood J.L., Vígh L. (2015). Membrane lipid therapy: modulation of the cell membrane composition and structure as a molecular base for drug discovery and new disease treatment. Prog. Lipid Res..

[4] Appelt C., Schrey A.K., Söderhäll J.A., Schmieder P. (2007). Design of antimicrobial compounds based on peptide structures. Bioorg. Med. Chem. Lett..

[5] Galdiero S., Falanga A., Berisio R., Grieco P., Morelli G., Galdiero M. (2015). Antimicrobial peptides as an opportunity against bacterial diseases. Curr. Med. Chem..

[6] Fjell C.D., Hiss J.A., Hancock R.E., Schneider G. (2012). Designing antimicrobial peptides: form follows function. Nat. Rev. Drug Discov..

[7] Finger S., Kerth A., Dathe M., Blume A. (2015). The efficacy of trivalent cyclic hexapeptides to induce lipid clustering in PG/PE membranes correlates with their antimicrobial activity. Biochim. Biophys. Acta, Biomembr..

[8] Ambrosio R.L., Rosselló C.A., Casares D., Palmieri G., Anastasio A., Escribá P.V. (2022). The antimicrobial peptide 1018-K6 interacts distinctly with eukaryotic and bacterial membranes, the basis of its specificity and bactericidal activity. Int. J. Mol. Sci..

[9] Chan D.I., Prenner E.J., Vogel H.J. (2006). Tryptophan- and arginine-rich antimicrobial peptides: structures and mechanisms of action. Biochim. Biophys. Acta.

[10] Shai Y. (1999). Mechanism of the binding, insertion, and destabilization of phospholipid bilayer membranes by α-helical antimicrobial and cell non-selective membrane-lytic peptides. Biochim. Biophys. Acta.

[11] Chook C.Y.B., Chen F.M., Leung F.P., Chen Z.Y., Wong W.T. (2021). Potential of crocodile blood as a medication and dietary supplement: a systemic review. Clin. Exp. Pharmacol. Physiol..

[12] Theansungnoen T., Maijaroen S., Jangpromma N., Yaraksa N., Daduang S., Temsiripong T., Klaynongsruang S. (2016). Cationic antimicrobial peptides derived from Crocodylus siamensis leukocyte extract, revealing anticancer activity and apoptotic induction on human cervical cancer cells. Protein J..

[13] Khalid S., Schroeder C., Bond P.J., Duncan A.L. (2022). What have molecular simulations contributed to the understanding of Gram-negative bacterial cell envelopes?. Microbiology.

[14] Mortazavi S.H., Bozorgmehr M.R., Heravi M.M. (2020). Structure of the cyclic, cationic antimicrobial peptide (kkwwkf) in octanol solution: in silico approach. Indonesian Journal of Chemistry.

[15] de Oliveira E.C.L., da Costa K.S., Taube P.S., Lima A.H., Junior C.D.S.D.S. (2022). Biological membrane-penetrating peptides: computational prediction and applications. Front. Cell. Infect. Microbiol..

[16] Garrido N.M., Queimada A.J., Jorge M., Macedo E.A., Economou I.G. (2009). 1-Octanol/water partition coefficients of n-alkanes from molecular simulations of absolute solvation free energies. J. Chem. Theor. Comput..

[17] Liu Z., Clark A.E. (2021). An octanol hinge opens the door to water transport. Chem. Sci..

[18] MacCallum J.L., Tieleman D.P. (2002). Structures of neat and hydrated 1-octanol from computer simulations. J. Am. Chem. Soc..

[19] Ermondi G., Vallaro M., Saame J., Toom L., Leito I., Ruiz R., Caron G. (2021). Rifampicin as an example of beyond-rule-of-5 compound: ionization beyond water and lipophilicity beyond octanol/water. Eur. J. Pharmaceut. Sci..

[20] Abraham M.J., Murtola T., Schulz R., Páll S., Smith J.C., Hess B., Lindahl E. (2015). GROMACS: high performance molecular simulations through multi-level parallelism from laptops to supercomputers. SoftwareX.

[21] Lamiable A., Thévenet P., Rey J., Vavrusa M., Derreumaux P., Tufféry P. (2016). PEP-FOLD3: faster de novo structure prediction for linear peptides in solution and in complex. Nucleic Acids Res..

[22] Biswal M.R., Rai S., Prakash M.K. (2019). Molecular dynamics based antimicrobial activity descriptors for synthetic cationic peptides. J. Chem. Sci..

[23] Milletti F. (2012). Cell-penetrating peptides: classes, origin, and current landscape. Drug Discov. Today.

[24] Mirnejad R., Fasihi-Ramandi M., Behmard E., Najafi A., Moghaddam M.M. (2022). Antibacterial CM-11 peptide potency against the gram-positive and gram-negative bacterial membrane models: a molecular dynamics simulations study. Research Square.

[25] Nguyen M.T.H. (2022). Ionic strength and solution composition dictate the adsorption of cell-penetrating peptides onto phosphatidylcholine membranes. Langmuir.

[26] Vargas J.R., Stanzl E.G., Teng N.N., Wender P.A. (2014). Cell-penetrating, guanidinium-rich molecular transporters for overcoming efflux-mediated multidrug resistance. Mol. Pharm..

[27] Sun D., Forsman J., Woodward C.E. (2015). Evaluating force fields for the computational prediction of ionized arginine and lysine side-chains partitioning into lipid bilayers and octanol. J. Chem. Theor. Comput..

[28] Jindal M.H., Le C.F., Mohd Yusof M.Y., Sekaran S.D. (2014). Net charge, hydrophobicity, and specific amino acids contribute to the activity of antimicrobial peptides. J. Health Transl. Med..

[29] Rousseau F., Schymkowitz J., Serrano L. (2006). Protein aggregation and amyloidosis: confusion of the kinds?. Curr. Opin. Struct. Biol..

[30] Shepherd C.M., Vogel H.J., Tieleman D.P. (2003). Interactions of the designed antimicrobial peptide MB21 and truncated dermaseptin S3 with lipid bilayers: molecular-dynamics simulations. Biochem. J..

[31] Hess B. (1997). LINCS: a linear constraint solver for molecular simulations. J. Comput. Chem..

[32] Mahnam K., Zarean M., Ghobadi Z. (2024). Lasioglossin-1 peptide inhibits binding of spike protein of SARS-CoV-2 to ACE2 receptor: an in silico approach of some bee venom peptides. Mol. Simul..

[33] Schüttelkopf A.W., Van Aalten D.M. (2004). PRODRG: a tool for high-throughput crystallography of protein–ligand complexes. Acta Crystallogr D Biol Crystallogr.

[34] Bemporad D., Luttmann C., Essex J.W. (2004). Computer simulation of small molecule permeation across a lipid bilayer: dependence on bilayer properties and solute volume, size, and cross-sectional area. Biophys. J..

[35] Jurrus E. (2018). Improvements to the APBS biomolecular solvation software suite. Protein Sci..

[36] Amadei A., Linssen A.B., Berendsen H.J. (1993). Essential dynamics of proteins. Proteins: Struct., Funct., Bioinf..

[37] Sang P., Du X., Yang L., Meng Z., Liu S. (2017). Molecular motions and free-energy landscape of serine proteinase K in relation to its cold-adaptation: a comparative molecular dynamics simulation study and the underlying mechanisms. RSC Adv..

[38] Jenik M., Parra R.G., Leandro G.R., Radusky L.G., Turjanski A., Peter G. (2012). Protein frustratometer: a tool to localize energetic frustration in protein molecules. Nucleic Acids Res..

[39] Freiberger M.I., Ruiz-Serra V., Pontes C., Romero-Durana M., Galaz-Davison P., Ramírez-Sarmiento C.A., Schuster C.D., Marti M.A., Wolynes P.G., Ferreiro D.U., Parra R.G., Valencia A. (2023). Local energetic frustration conservation in protein families and superfamilies. Nat. Commun..

[40] Ferreiro D.U., Hegler J.A., Komives E.A., Wolynes P.G. (2007). Localizing frustration in native proteins and protein assemblies. Proc. Natl. Acad. Sci. USA.

[41] Shazely B.E.l., Yu G., Johnston P.R., Rolff J. (2020). Resistance evolution against antimicrobial peptides in Staphylococcus aureus alters pharmacodynamics beyond the MIC. Front. Microbiol..

[42] Green M.R., Sambrook J. (2001). Molecular cloning: a laboratory manual: cold spring harbor laboratory cold spring harbor, NY. https://cshprotocols.cshlp.org.

[43] Huuskonen J., Livingstone D.J., Manallack D.T. (2008). Prediction of drug solubility from molecular structure using a drug-like training set. SAR QSAR Environ. Res..

[44] Fernández-Vidal M., Jayasinghe S., Ladokhin A.S., White S.H. (2007). Folding amphipathic helices into membranes: amphiphilicity trumps hydrophobicity. J. Mol. Biol..

[45] Games P.A., Howell J.F. (1976). Pairwise multiple comparison procedures with unequal N's and/or variances: a Monte Carlo study. Journal of Educational and Behavioural Statistics.

[46] Dorairaj S., Allen T.W. (2007). On the thermodynamic stability of a charged arginine side chain in a transmembrane helix. Proc. Natl. Acad. Sci. USA.

[47] R.M. Sabuti, M.R. Bozorgmehr, A. Morsali, Molecular dynamics simulations on the heterocyclic cyclodecapeptide and its linear analogous in water and octanol solvents. J. Mol. Liq.. 229, (217) 583-590. DOI: 10.1016/j.molliq.2016.11.118.

[48] Frazee N., Billlings K.R., Mertz B. (2024). Gaussian accelerated molecular dynamics simulations facilitate prediction of the permeability of cyclic peptides. PLoS One.

[49] Chen Y., Mant C.T., Farmer S.W., Hancock R.E., Vasil M.L., Hodges R.S. (2005). Rational design of α-helical antimicrobial peptides with enhanced activities and specificity/therapeutic index. J. Biol. Chem..

[50] Jahanbin F., Bozorgmehr M.R., Morsali A., Beyramabadi S.A. (2019). The effect of different alcohols on the Asp23-Lys28 and Asp23-Ala42 salt bridges of the most effective peptide in Alzheimer's disease: molecular dynamics viewpoints. J. Mol. Graph. Model..

[51] Yeaman M.R., Yount N.Y. (2003). Mechanisms of antimicrobial peptide action and resistance. Pharmacol. Rev..

[52] Malanovic N., Lohner K. (2016). Antimicrobial peptides targeting gram-positive bacteria. Pharmaceuticals.

[53] Torrent M., Andreu D., Nogués V.M., Boix E. (2011). Connecting peptide physicochemical and antimicrobial properties by a rational prediction model. PLoS One.

[54] White S.H. (2003). Translocons, thermodynamics, and the folding of membrane proteins. FEBS Lett..

