# Supplementary material for: Improving the antimicrobial activity of RP9 peptide through theoretical and experimental investigation
Source: Biochem Biophys Rep. 2025 Feb 15;41:101953. doi: 10.1016/j.bbrep.2025.101953 (PMC11872504; doi:10.1016/j.bbrep.2025.101953)
Supplement: Multimedia component 1 [file mmc1.pdf]

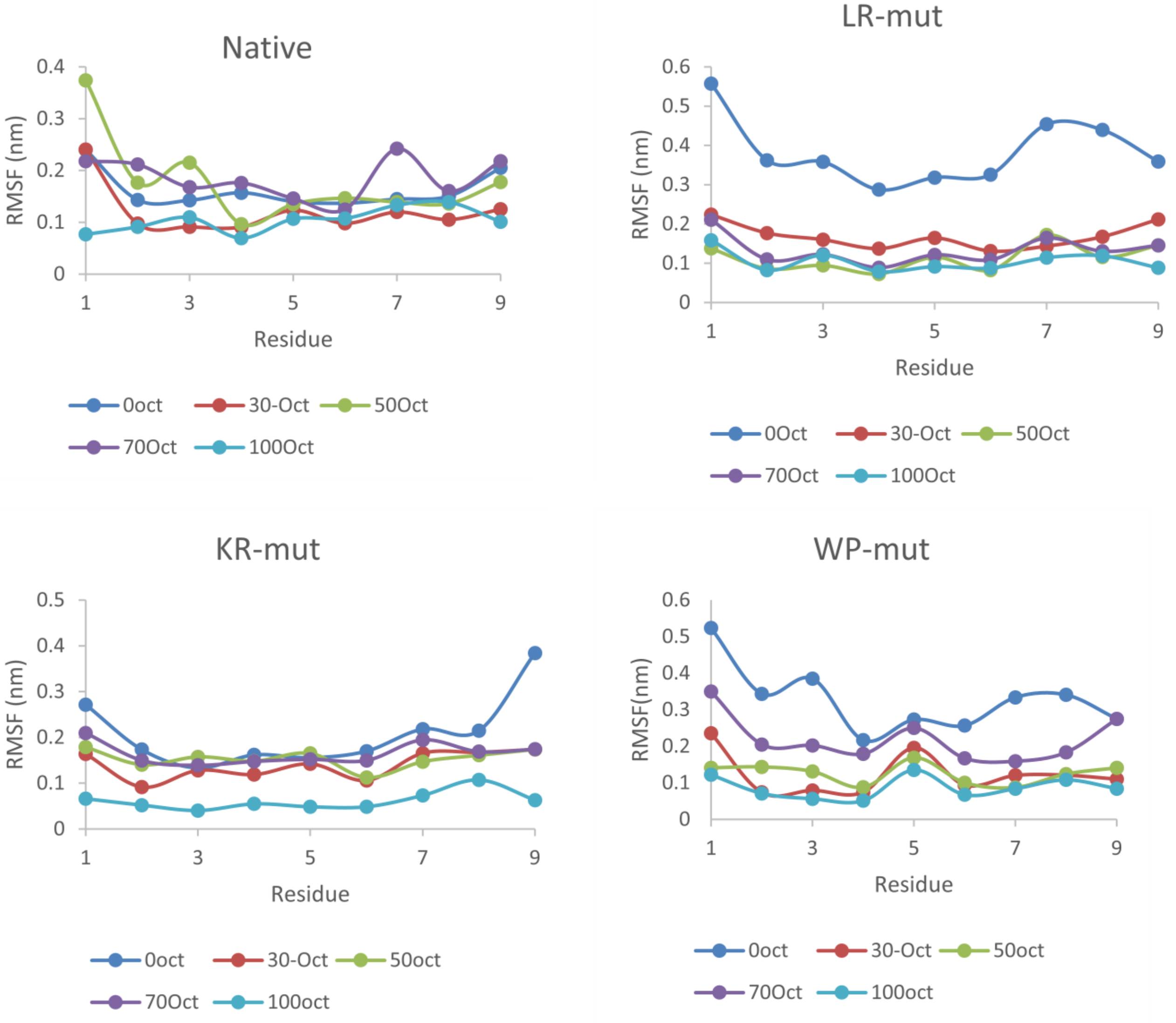

Figure S1. The RMSF per residue of all peptides in all octanol concentrations during the last 50 ns of MD simulation.

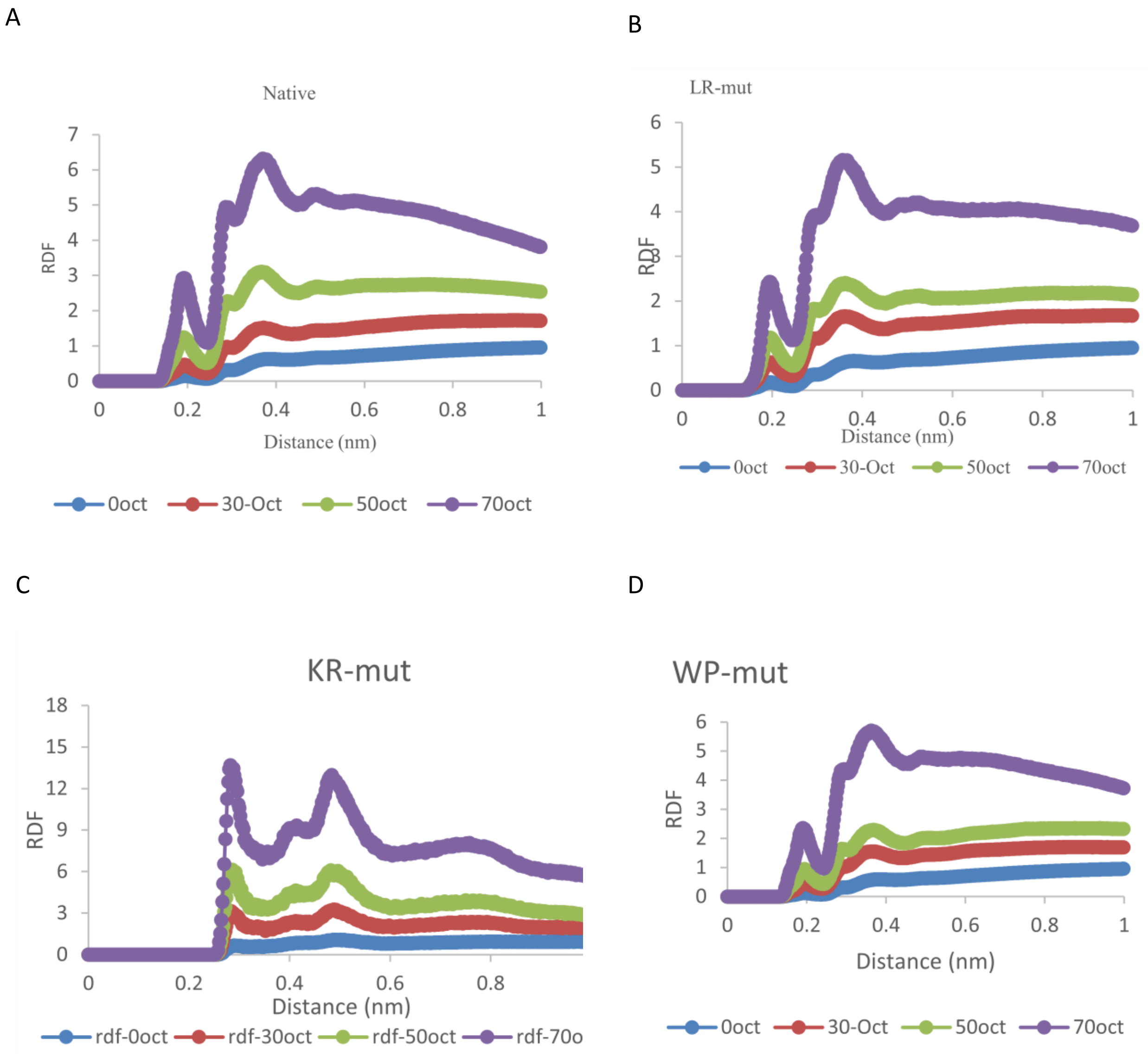

Figure S2. The RDF plot of the water's oxygen atoms surrounding the NE atom of the Arg1 of all peptides at different octanol concentrations.

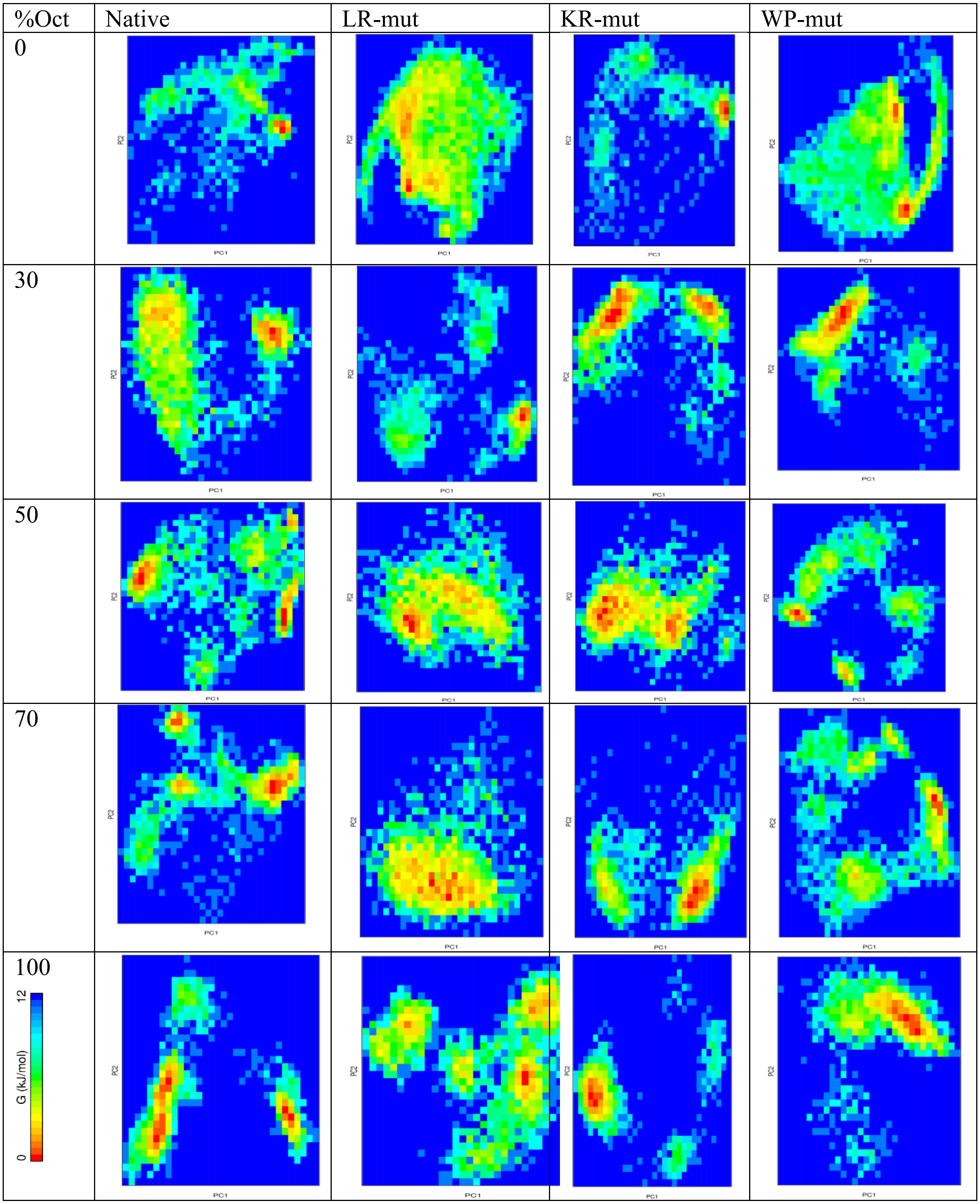

Figure S3. The Gibbs free energy landscape of peptides for PC1 versus PC2 in different octanol concentrations during 200 ns of MDS. The free energy ranges were between 0 (red color) to about 12 (blue color)

A

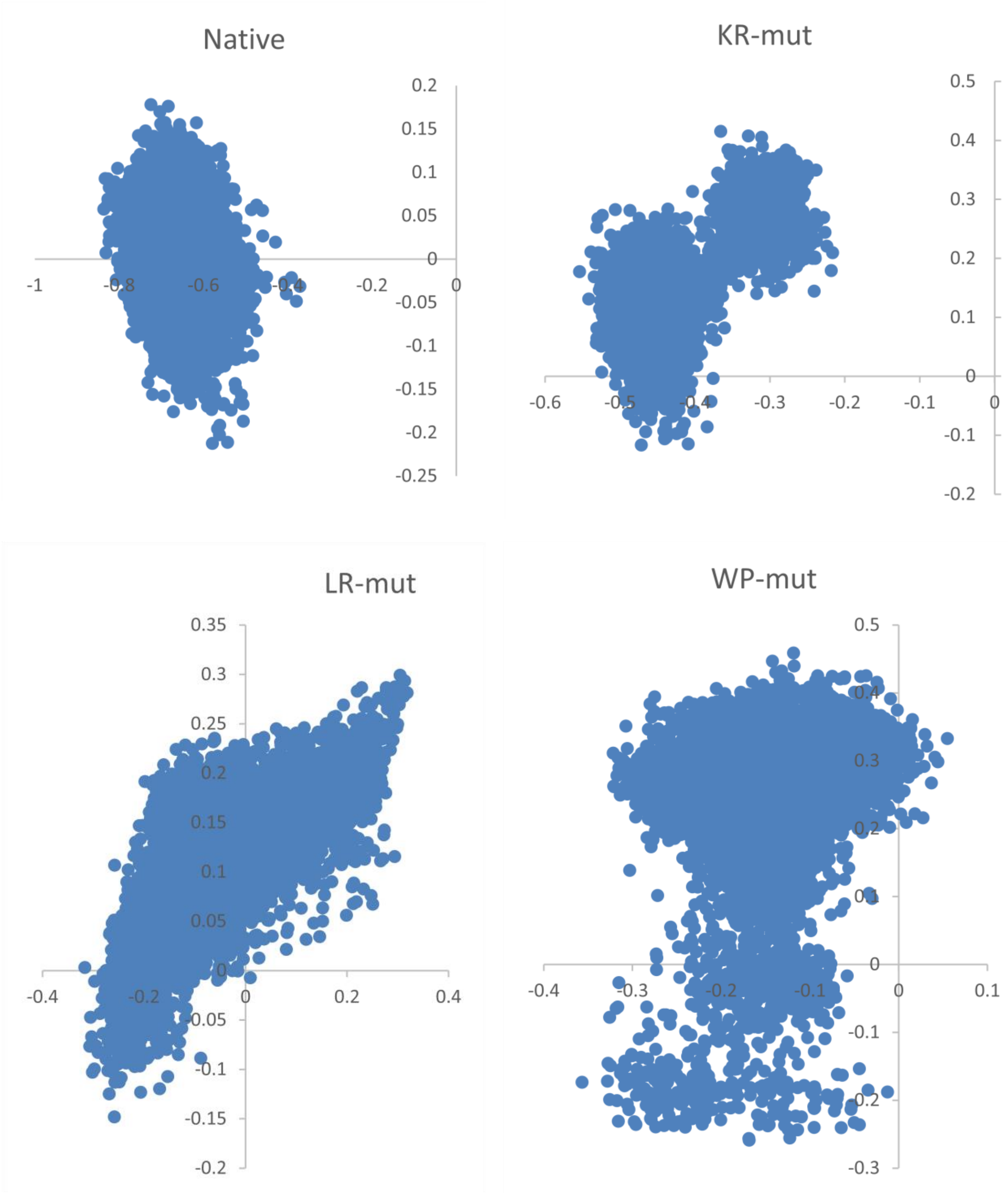
Native
0.2
0.15
0.1
0.05
0
-0.05
-0.1
-0.15
-0.2
-0.25
-1
-0.8
-0.6
-0.4
-0.2
0
KR-mut
0.5
0.4
0.3
0.2
0.1
0
-0.1
-0.2
-0.6
-0.5
-0.4
-0.3
-0.2
-0.1
0
LR-mut
0.35
0.3
0.25
0.2
0.15
0.1
0.05
0
-0.05
-0.1
-0.15
-0.2
-0.4
-0.2
0
0.2
0.4
WP-mut
0.5
0.4
0.3
0.2
0.1
0
-0.1
-0.2
-0.3
-0.4
-0.3
-0.2
-0.1
0
0.1

B

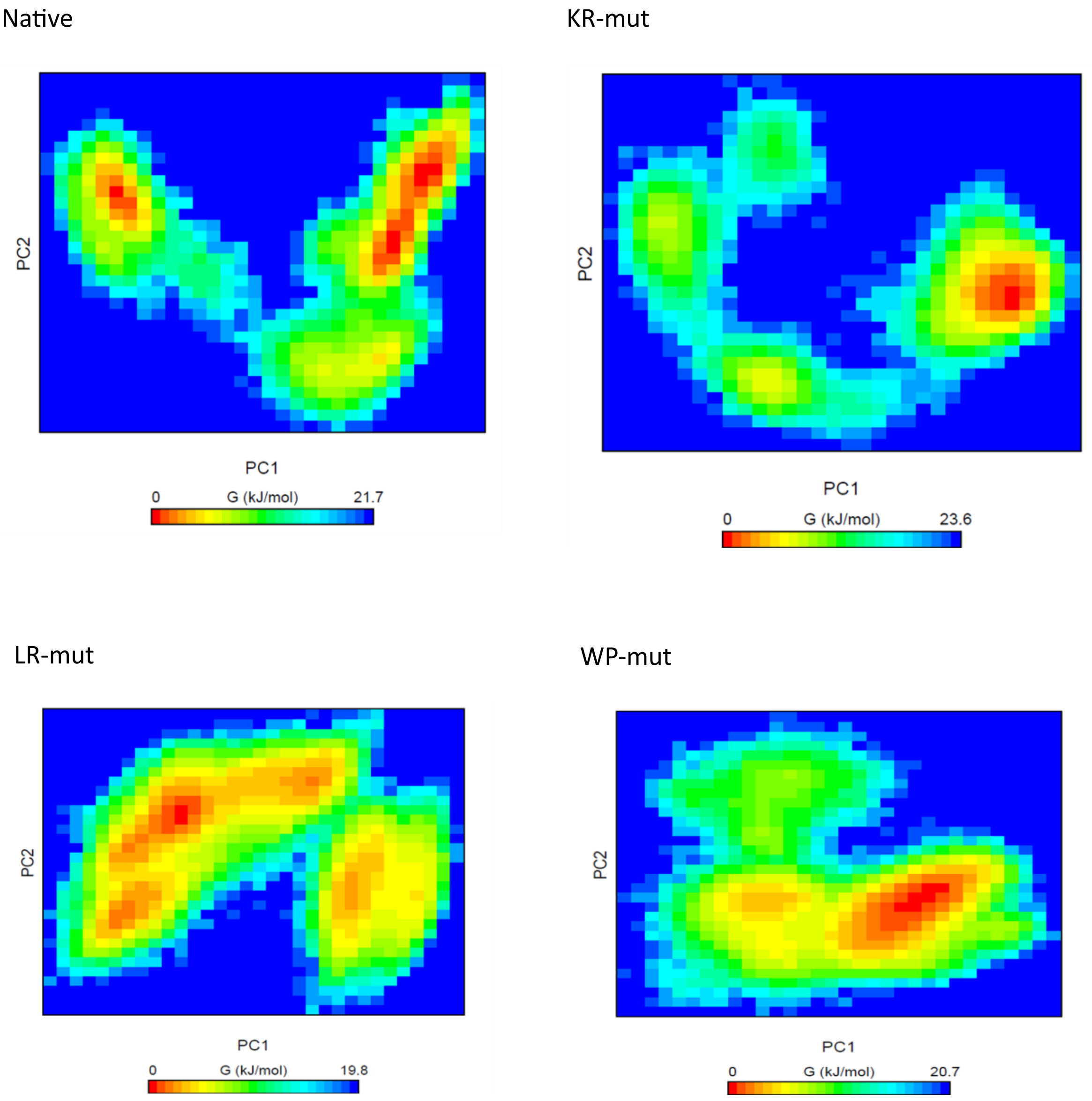

Figure S4. The PC1-PC2 plot of concatenated trajectories of each peptide (A) and their free energy landscapes (B) during 200 ns MDS.

Nattive0

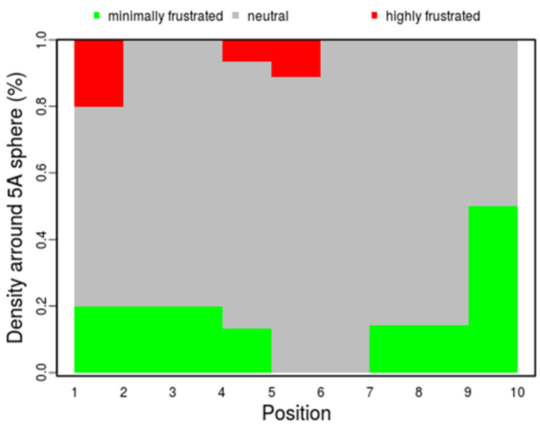

Nattive30

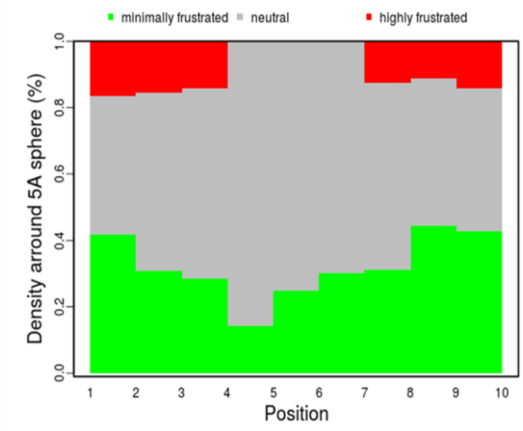

Native50

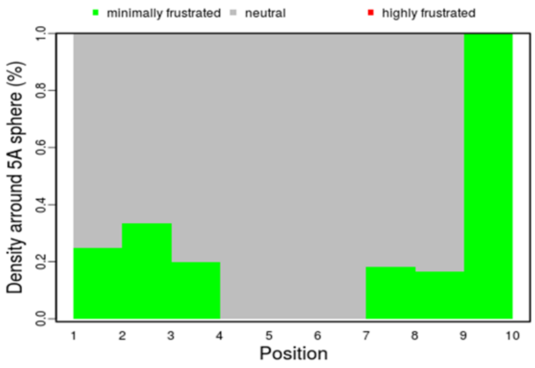

Native70

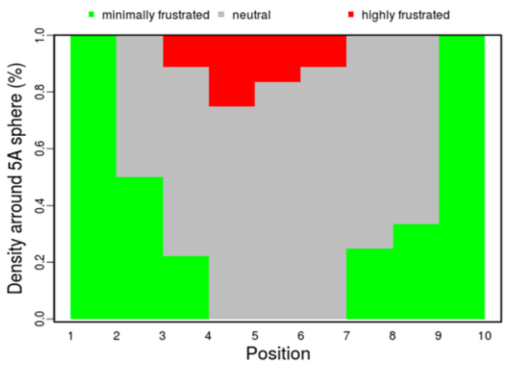

Native100

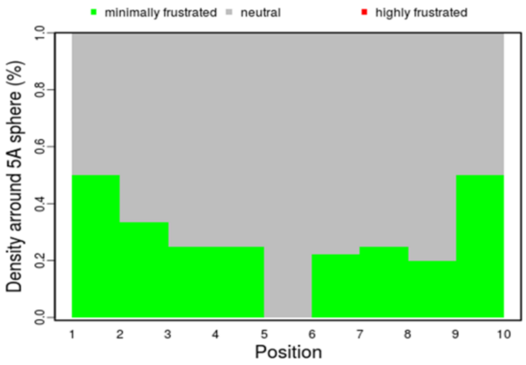

WP0

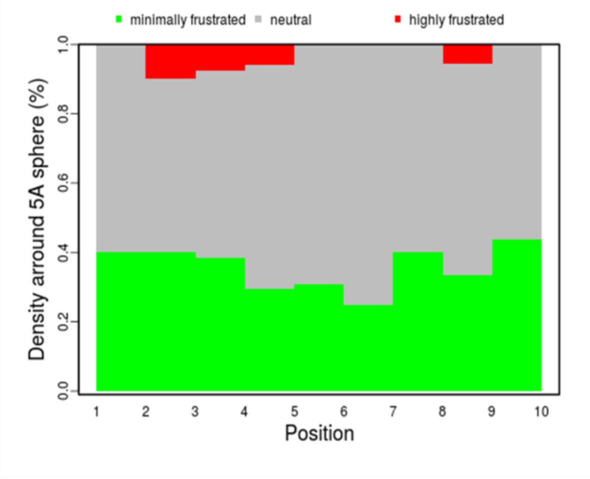

WP30

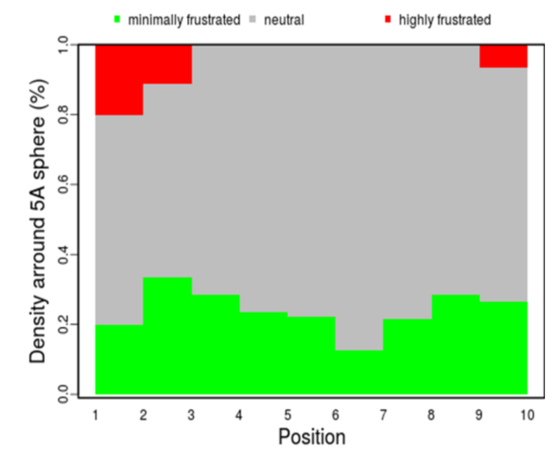

WP50

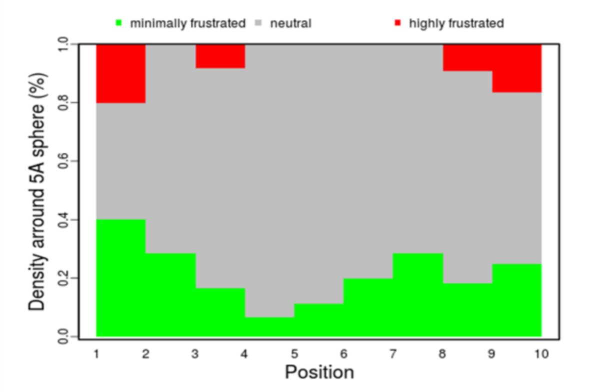

WP70

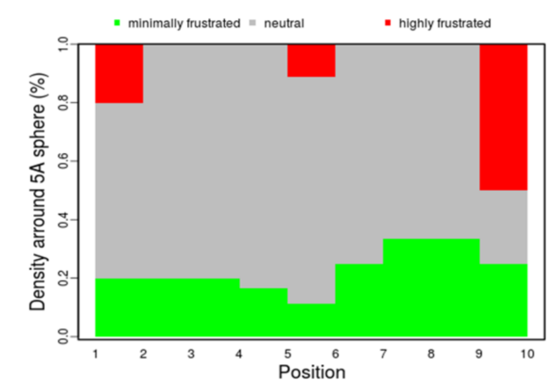

WP100

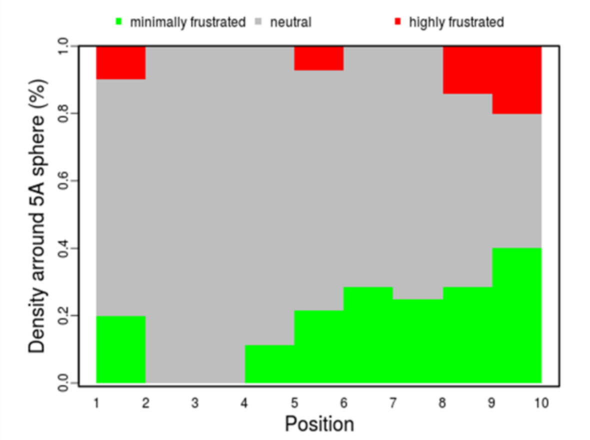

LR0

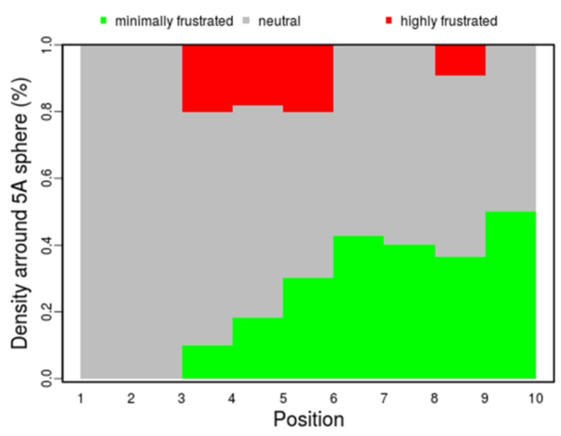

LR30

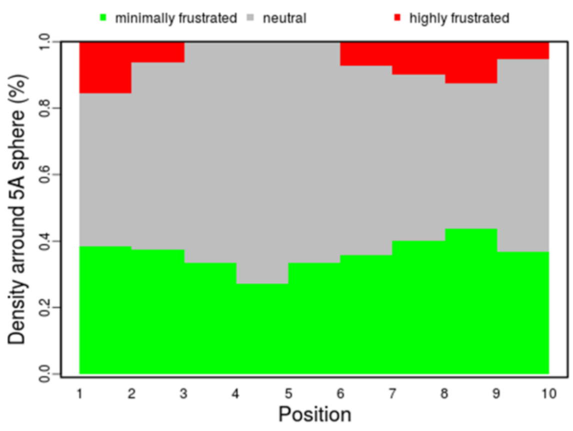

LR50

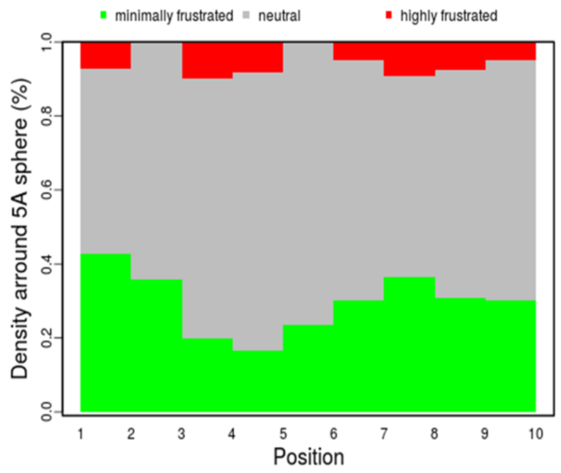

LR70

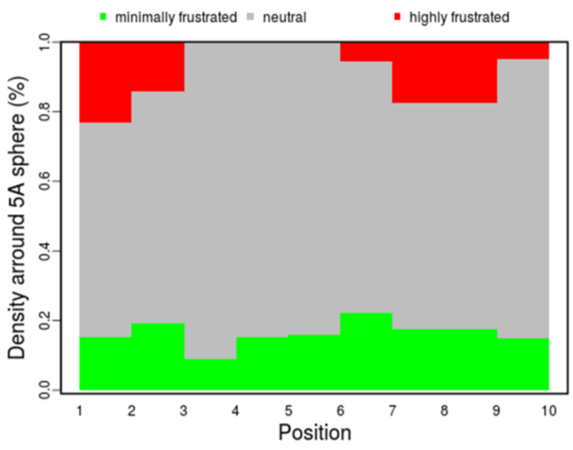

LR100

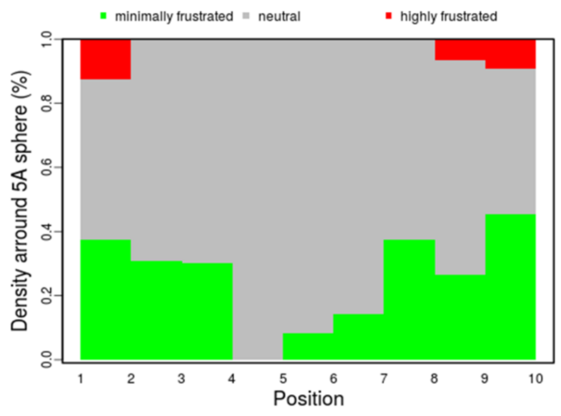

KR0

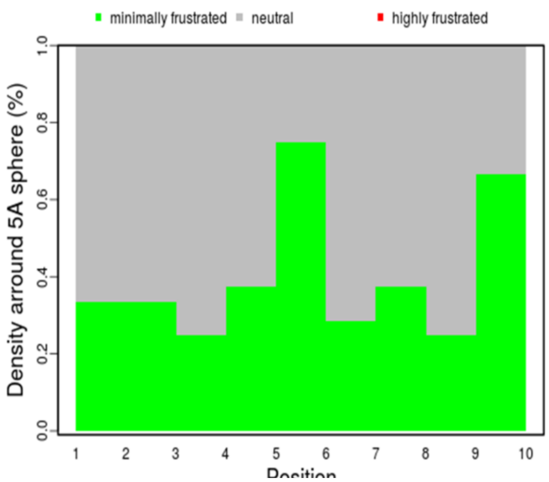

KR30

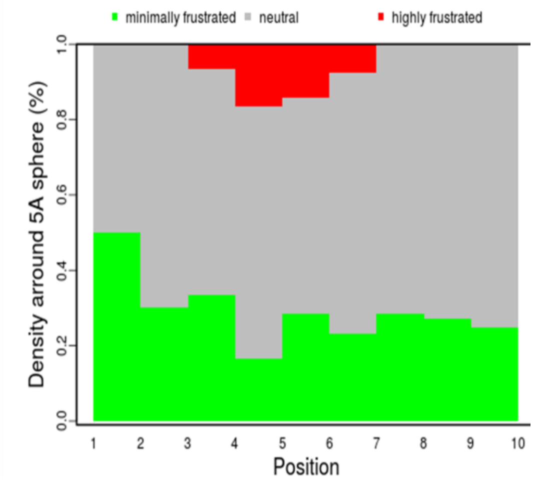

KR50

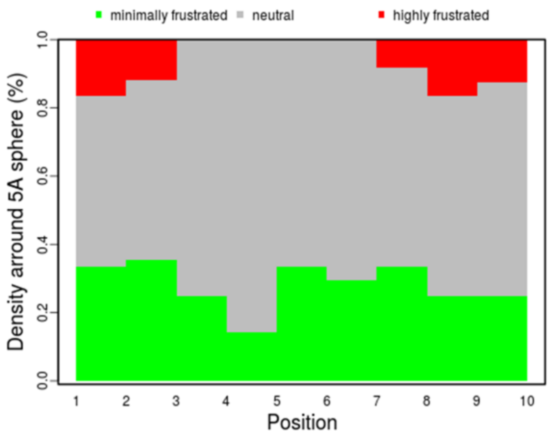

KR70

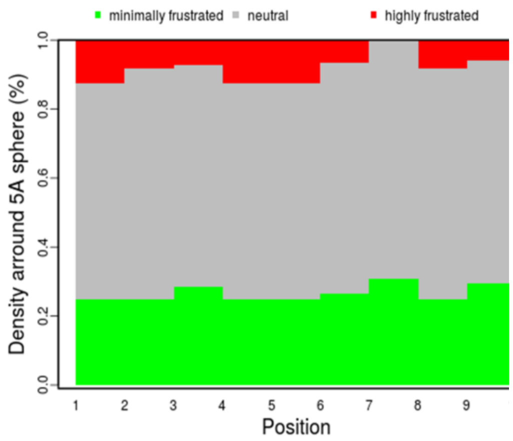

KR100

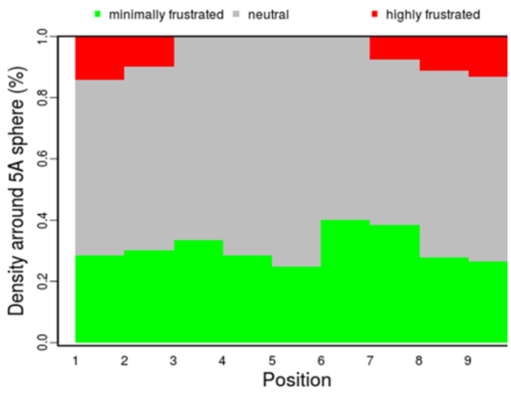

Figure S5. The single residue frustration configurational map of peptides in all octanol concentration (high frustrated residues in red and minimal frustrated residues in green color).

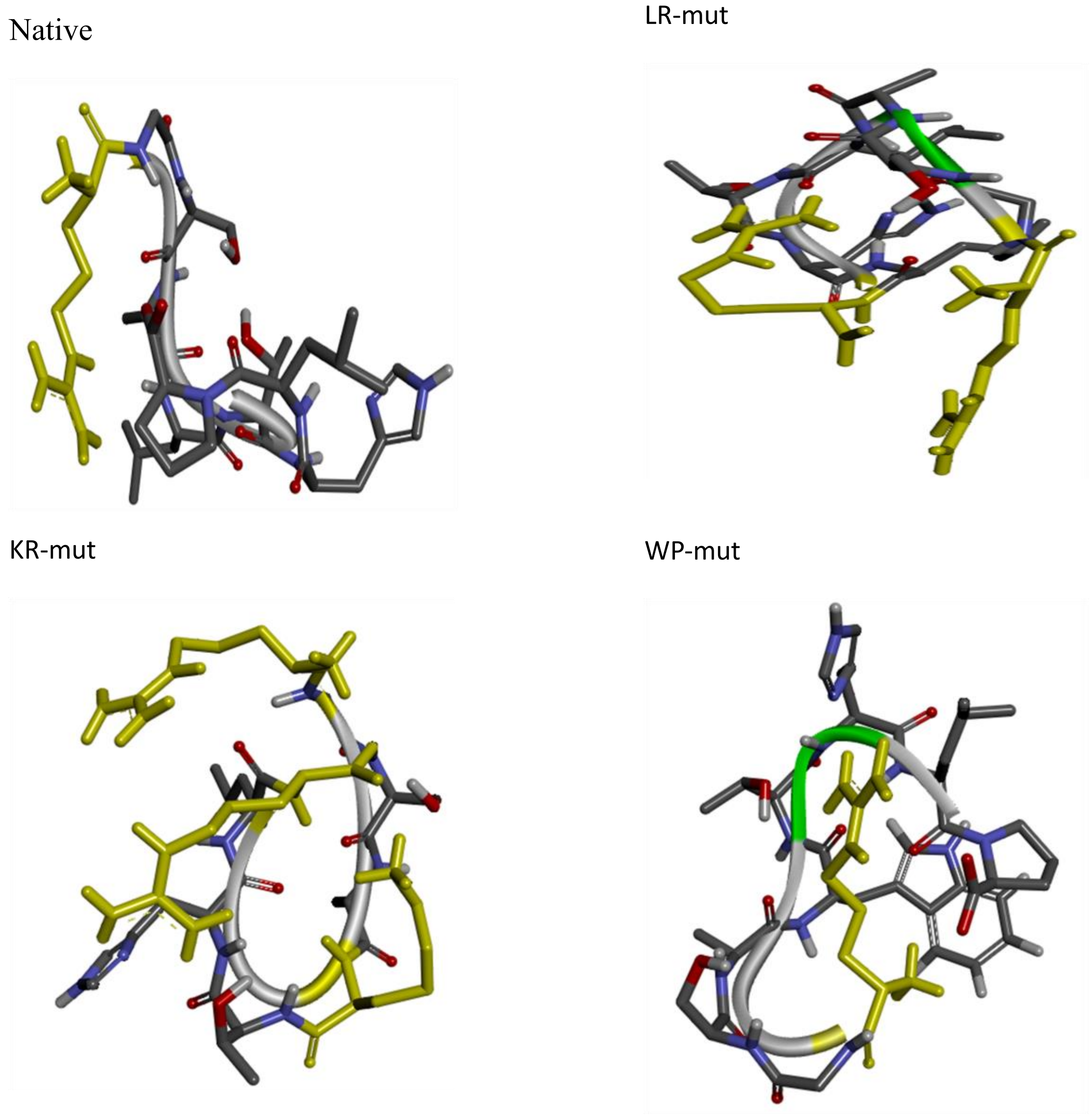

Figure S6. The final structure of all peptides after 200 ns MDS. The side chains of Arg and Lys residues in all peptides stray out of the peptides and colored in yellow.

Table S1. The first eigenvalues (FE) and second (SE) eigenvalues of PCA analysis of peptides in different octanol concentrations during 200 ns MDS.

%Octanol	Native		LR-mut		KR-mut		WP-mut	
	FE	SE	FE	SE	FE	SE	FE	SE
0	0.225	0.127	0.262	0.127	0.177	0.053	0.347	0.144
30	0.052	0.030	0.250	0.076	0.092	0.018	0.050	0.016
50	0.643	0.050	0.018	0.006	0.042	0.006	0.157	0.085
70	0.100	0.044	0.017	0.006	0.079	0.012	0.127	0.085
100	0.111	0.013	0.044	0.012	0.018	0.005	0.009	0.007
